# The *Bacillus* BioBrick Box 2.0: expanding the genetic toolbox for the standardized work with *Bacillus subtilis*

**DOI:** 10.1038/s41598-017-15107-z

**Published:** 2017-11-08

**Authors:** Philipp F. Popp, Mona Dotzler, Jara Radeck, Julia Bartels, Thorsten Mascher

**Affiliations:** 1 Institute of Microbiology, Technische Universität (TU) Dresden, Dresden, Saxony 01062 Germany; 2 Institute of Microbiology, Ludwig-Maximilians-Universität (LMU) München, Planegg-Martinsried, Bavaria, 82152 Germany; 3 Present Address: Institute of Microbiology, TU Dresden, Dresden, Saxony 01062 Germany

## Abstract

Standardized and well-characterized genetic building blocks allow the convenient assembly of novel genetic modules and devices, ensuring reusability of parts and reproducibility of experiments. In the first *Bacillus subtilis*-specific toolbox using the BioBrick standard, we presented integrative vectors, promoters, reporter genes and epitope tags for this Gram-positive model bacterium. With the *Bacillus* BioBrick Box 2.0, we significantly expand the range of our toolbox by providing new integrative vectors, introducing novel tools for fine-tuning protein expression, and carefully evaluating codon-adapted fluorescence proteins in *B. subtilis*, which cover the whole spectrum of visible light. Moreover, we developed new reporter systems to allow evaluating the strength of promoters and ribosome binding sites. This well-evaluated extension of our BioBrick-based toolbox increases the accessibility of *B. subtilis* and will therefore promote the use of this model bacterium and biotechnological workhorse as a host for fundamental and applied Synthetic Biology projects.

## Introduction

One key feature of Synthetic Biology (SynBio) is the implementation of engineering approaches, such as abstraction, modularity and standardization into biological procedures directly at the DNA level. Genetic elements, such as promoters or open reading frames, are considered individually and independently of genomic context, which postulates them as *parts* with a predictable function and biological behavior^1–4^. One of the first collections of *parts* fulfilling biological standardization, was the *Registry of Standard Biological Parts* (partsregistry), which at present harbors more than 12.000 genetic *parts* ^5^. The majority of these so-called BioBricks (*parts*) were contributed by teams participating in the annual *international Genetically Engineered Machines* (iGEM) student competition^6^. Each *part* is provided with background information, design aspects and *part* properties, including experimental data^5^. All submitted *parts* have to fulfill the requirements of the genetic assembly standard described in the *request for comments 10* (RFC10)^7^. The standard defines four type II restriction endonucleases flanking each *part* and prohibits their occurrence inside the *parts* DNA sequence. The assembly process of the BioBrick standard is idempotent because the combination of two *parts* will preserve the pre- and suffix of the standard after the assembly and removes the restriction sites in between the *parts*. Thus, the newly formed *composite part* (e.g. a fusion of promoter to a gene of interest) can again be recombined with any other BioBrick in a second round of assembly, using the same restriction enzymes^8^. While the RFC10 standard thereby enables an infinite re-plugging of *parts*, it only allows transcriptional fusions, due to the formation of a scar of eight nucleotides between assembled parts. In order to overcome this limitation, the RFC25 standard was established^9^. It introduced two additional restriction sites together with the original BioBrick standard pre- and suffix, which form a six nucleotide scar upon assembly. Thereby it allows the construction of translational fusions, such as the in frame combination of a protein-coding sequence with a fluorescent protein tag.

While the BioBrick standard is organism-independent, the vast majority of *parts* submitted to the partsregistry are designed for the work with *Escherichia coli* ^3,10^. The number of *parts* available for other microorganisms, such as *Bacillus subtilis*, is low and many lack good documentation and evaluation.

*B. subtilis* is the best-studied Gram-positive microorganisms, and a model bacterium for studying bacterial differentiation (e.g. endospore formation) and phenotypic heterogeneity. Its ability to become naturally competent makes *B. subtilis* an organism with easily tractable genetics^11–13^. The GRAS (generally recognized as safe) status and secretory capacity made *B. subtilis* a preferred host of choice for big scale production of secreted proteins, such as lipases, proteases and amylases, highlighting the industrial relevance of this bacterium^14–18^.

In 2013, we introduced the first BioBrick toolbox for *B. subtilis*, providing a collection of well-evaluated integrative vectors, promoters, as well as epitope tags for the standardized work with *B. subtilis* ^19^. This toolbox was very well received and is in high demand, as indicated by over 12.000 views of the original article and over 500 *part* requests from the Bacillus Genetic Stock Center^20^ (BGSC) since July 2013 (personal communication with Dr. Daniel Zeigler; director of the BGSC). This success motivated us for the extension presented in this article.

Here, we focused on providing new BioBrick-vectors as addition to our previous toolbox (Table 1). We expand our existing collection of empty integrative vectors by equipping them with new antibiotic resistance cassettes. In addition, we created integrative and replicative expression vectors, which harbor one of three different inducible promoters upstream of the multiple cloning site (MCS). Furthermore, we developed three novel screening vectors: two for the investigation of ribosome binding site libraries and one for screening promoter libraries. Finally, we optimized and evaluated seven different fluorescent proteins (FPs) covering the whole spectrum of light for the use in *B. subtilis*.

**Table 1:** Tools provided in the BioBrick Box 1.0 and 2.0.

BioBrick^1^	Description^2^	Source	BGSC^3^	Ref.
**BioBrick Box 1.0**				
**Vectors**				
pBS1C	empty vector, integration at *amyE*, amp^r^, cm^r^	pDG1662-derivative	ECE257	19
pBS2E	empty vector, integration at *lacA*, amp^r^, mls^r^	pAX01-derivative	ECE258	19
pBS4S	empty vector, integration at *thrC*, amp^r^, spec^r^	pDF1731-derivative	ECE259	19
pBS1C*lacZ*	*lacZ*-reporter vector, integration at *amyE*, amp^r^, cm^r^	pAC6-derivative	ECE260	19
pBS3C*lux*	*lux*-reporter vector, integration at *sacA*, amp^r^, cm^r^	pAH328-derivative	ECE261	19
**BioBrick Box 2.0**				
**Vectors**				
pBS1E	empty vector, integration at *amyE*, amp^r^, mls^r^	pBS1C-derivative	ECE730	This study
pBS1K	empty vector, integration at *amyE*, amp^r^, kan^r^	pBS1C-derivative	ECE731	This study
pBS0E	empty vector, replicative ori1030, amp^r^, mls^r^	pGP380-derivative	ECE732	This study
pBS3K*lux*	*lux*-reporter vector, integration at *sacA*, amp^r^, kan^r^	pBS3C-derivative	ECE733	This study
pBS3E*lux*	*lux*-reporter vector, integration at *sacA*, amp^r^, mls^r^	pBS3C-derivative	ECE734	This study
pBS3K*catlux*	*lux*-reporter vector, *cat*, integration at *sacA*, amp^r^, kan^r^	pBS3C-derivative	ECE735	This study
pBS3E*catlux*	*lux*-reporter vector, *cat*, integration at *sacA*, amp^r^, mls^r^, cm^r^	pBS3C-derivative	ECE736	This study
pBS1C*αlacZ*	*lacZ*-reporter vector (exch. RBS-site), integration at *amyE*, *lacZα*, amp^r^, cm^r^	pBS3C-derivative	ECE737	This study
pBS3C*αlux*	*lux*-reporter vector (exch. RBS-site of *luxA*), integration at s*acA*, *lacZα*, amp^r^, cm^r^	pBS1C-derivative	ECE738	This study
pBS2EP_*xylA* (V2)_	empty vector, integration at *lacA*, P_*xylA*_ upstream of MCS, amp^r^, mls^r^	pBS2E-derivative	ECE739	This study
pBS2EP_*liaI* (V2)_	empty vector, integration at *lacA*, P_*liaI*_ upstream of MCS, amp^r^, mls^r^	pBS2E-derivative	ECE740	This study
pBS2EXylRP_*xylA* (V2)_	empty vector, integration at *lacA*, XylR-P_*xylA*_ upstream of MCS, amp^r^, mls^r^	pBS2E-derivative	ECE741	This study
pBS0EP_*liaI* (V2)_	empty vector, ori1030, P_*liaI*_ upstream of MCS, amp^r^, mls^r^	pBS0E-derivative	ECE742	This study
pBS0EXylRP_*xylA* (V2)_	empty vector, ori1030, XylR-P_*xylA*_ upstream of MCS, amp^r^, mls^r^	pBS0E-derivative	ECE743	This study
**Fluorescent proteins**		**Ex/Em**		
mTagBFP	codon usage for *E. coli*	399**/**465	ECE744	60
mTagBFP_Bsu	codon optimized for *B. subtilis*	399**/**465	ECE745	This study
eCFP_Bsu	codon optimized for *B. subtilis*	449**/**479	ECE746	61
sfGFP_Spn	codon optimized for *S. pneumoniae* (RFC10 and RFC25)	481**/**511	ECE747/ECE748	62
GFPmut1	codon usage for *A. victoria*	483**/**513	ECE749	46
GFPmut1 (LT)	codon optimized for *B. subtilis* (algorithm used from LifeTech)	483**/**513	ECE750	This study
mEYFP	codon usage for *E. coli*	500**/**530	ECE751	61
mEYFP_Bsu	codon optimized for *B. subtilis*	500**/**530	ECE752	This study
SYFP2	codon usage for *E. coli* (RFC10 and RFC25)	500**/**530	ECE753/ECE754	63
mCherry	codon usage for *E. coli*	585**/**615	ECE755	64
mCherry_Bsu	codon optimized for *B. subtilis* (RFC10 and RFC25)	585**/**615	ECE756/ECE757	This study

^1^Nomenclature: p = plasmid, BS = *B. subtilis*, the number refers to the integration locus: 1 = *amyE*, 2 = *sacA* and 3 = *lacA*, whereas 0 stands for replicative; and the last letter codes for the resistance in *B. subtilis*: C = chloramphenicol (mediated by *cat*), E = MLS (mediated by *erm*: specifies resistance to macrolid-, linkosamid- and streptogramin B- antibiotics, if induced by erythromycin) and K = kanamycin (mediated by *kan*). The abbreviations in italics refer to the functional part of the reporter vectors: *lacZ* for the β-galactosidase *lacZ*, *lux* represents the *luxABCDE* operon mediating luminescence and *catlux* stands for the *cat* gene transcriptionally fused to the *luxABCDE* operon.

^2^Amp^r^, ampicillin resistance; cm^r^, chloramphenicol resistance; kan^r^, kanamycin resistance; spc^r^, spectinomycin resistance; mls^r^, erythromycin-induced resistance to macrolid-, linkosamid- and streptogramin B- antibiotics (MLS); cat, RBS and gene for chloramphenicol resistance; MCS, multiple cloning site.

^3^The Bacillus BioBrick Box 2.0 plasmids and part sequences are available at the BGSC (http://bgsc.org).

## Results and Discussion

### Empty vectors of the *Bacillus* BioBrick Box 2.0 with new combinations of resistance markers

**Figure 1:**
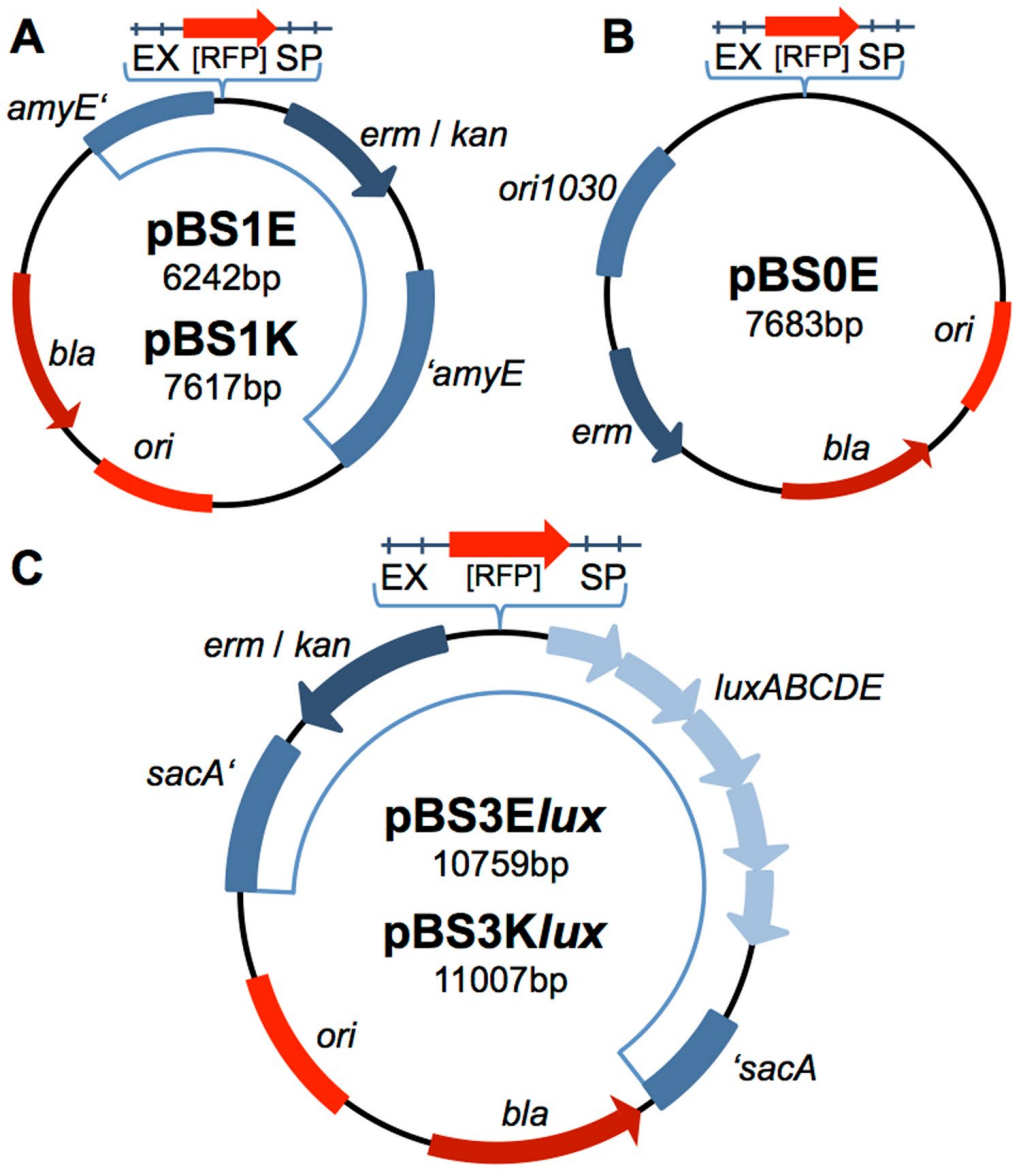
New empty and reporter vectors in RFC10 standard. Red parts indicate features for cloning in *E. coli*: the *bla* gene mediating resistance against ampicillin, the origin of replication (ori), and the multiple cloning site (MCS) which contains a gene encoding the red fluorescent protein (RFP) for red/white screening. In blue, *B. subtilis*-specific parts are depicted: the dark blue arrow represents the resistance cassette. (**A**,**C**) The boxes represent flanking homology regions used as integration sites for double homologous recombination into the *B. subtilis* chromosome. (**B**) The box represents the *B. subtilis*-specific origin of replication (ori) and the light blue arrows show the reporter operon *luxABCDE* encoding the *Photorhabdus luminescence* luciferase and accompanying enzymes for substrate generation and recycling. (**A**) The two empty vectors pBS1E and pBS1K integrate into the *amyE* locus and confer resistance either to macrolide, lincosamide and streptogramin B antibiotics (MLS) if induced by erythromycin (pBS1E, mediated by *erm*) or kanamycin (pBS1K, mediated by *kan*), respectively. **(B)** The first replicative vector in our toolbox, pBS0E, is equipped with ori1030 and confers resistance against MLS. **(C)** The reporter vectors encode luciferase (*luxABCDE*), intergrate into the *sacA* locus and confer resistance either against MLS (pBS3E*lux*) or kanamycin (pBS3K*lux*).

The first step in expanding the existing BioBrick box was to generate new integrative vectors by switching the *B. subtilis* specific antibiotic resistance cassettes of their original backbones^19^. For this purpose, we chose two frequently used vectors of our previous toolbox, the empty integrative backbone pBS1C and the luciferase reporter vector pBS3C*lux*. The vector pBS1C provides resistance against chloramphenicol in *B. subtilis* and integrates into the *amyE* locus, encoding the α-amylase. The resulting disruption leads to a loss of this enzymatic activity, thereby making it a vector easy to screen for by performing a starch test for positive integration events^19^. Unfortunately, the reporter vector pBS3C*lux* also provides chloramphenicol resistance, which prevents combining these two regularly used vectors in one strain. To overcome this limitation, we exchanged the chloramphenicol acetyl transferase in both cases for either the MLS (macrolide-lincosamide-streptogramin B) or kanamycin resistance cassette. The resulting new empty and reporter vectors, pBS1E/pBS1K and pBS3Elux/pBS3Klux, respectively (Fig. 1A and C) were evaluated by comparing them with the corresponding original backbones.

In case of pBS1E/K we cloned *lacZ* under the control of the constitutive promoter P_*veg*_ into the MCS and performed β-galactosidase assays (Supplemental Figure S2). Strains carrying one of the three vectors gave rise to ~40 Miller units when measured during exponential phase with no significant difference in growth nor for the measured β-galactosidase activity between the strains. Strains carrying the empty vectors were used as a control and had a residual activity of less than 1 Miller unit.

For the evaluation of the reporter vectors pBS3E*lux*/K*lux*, we inserted the constitutive promoters P_*veg*_ or P_*lepA*_ into the MCS and measured luciferase activity of the resulting *B. subtilis* strains. When compared with the output of the original backbones, no effects of changing the antibiotic resistances were observed on the functionality of the vectors (Supplemental Figure S2). All strains grew at normal rates and displayed promoter activities of 5*10^5^-5*10^6^ (P_*veg*_), or 10^5^-10^6^ relative luminescence units (RLU)/OD_600_ (P_*lepA*_), respectively. For pBS3E*lux*/K*lux*, minor differences in luciferase activities were observed in stationary phase (Supplemental Figure S1), however they were not significant.

Taken together, the exchange of resistance markers of pBS1C/E/K and pBS3C/E/K*lux* did not interfere with the measured output. The new vectors come especially in hand when there is a need of inserting several vectors into one single genome, allowing a higher flexibility in their combination, due to the alternative resistance cassettes.

We also adapted the empty backbone vector pGP380^21^ to the BioBrick standard, resulting in pBS0E, the first replicative *B. subtilis* vector in our toolbox (Fig. 1B). For this purpose, we first removed restriction enzyme sites, which interfered with the requirements of the RFC10 standard, followed by the insertion of an RFP cassette into the MCS (see methods for detailed construction). The medium copy number plasmid (15–25 per cell) will be particularly useful to overcome bottlenecks in protein overproduction generated by the limited copy number of integrative vectors, as has been observed previously^22,23^. To accommodate this need, we decided to also develop replicative expression vectors based on pBS0E, as described in the following section.

### A vector suite for inducible gene expression

**Figure 2:**
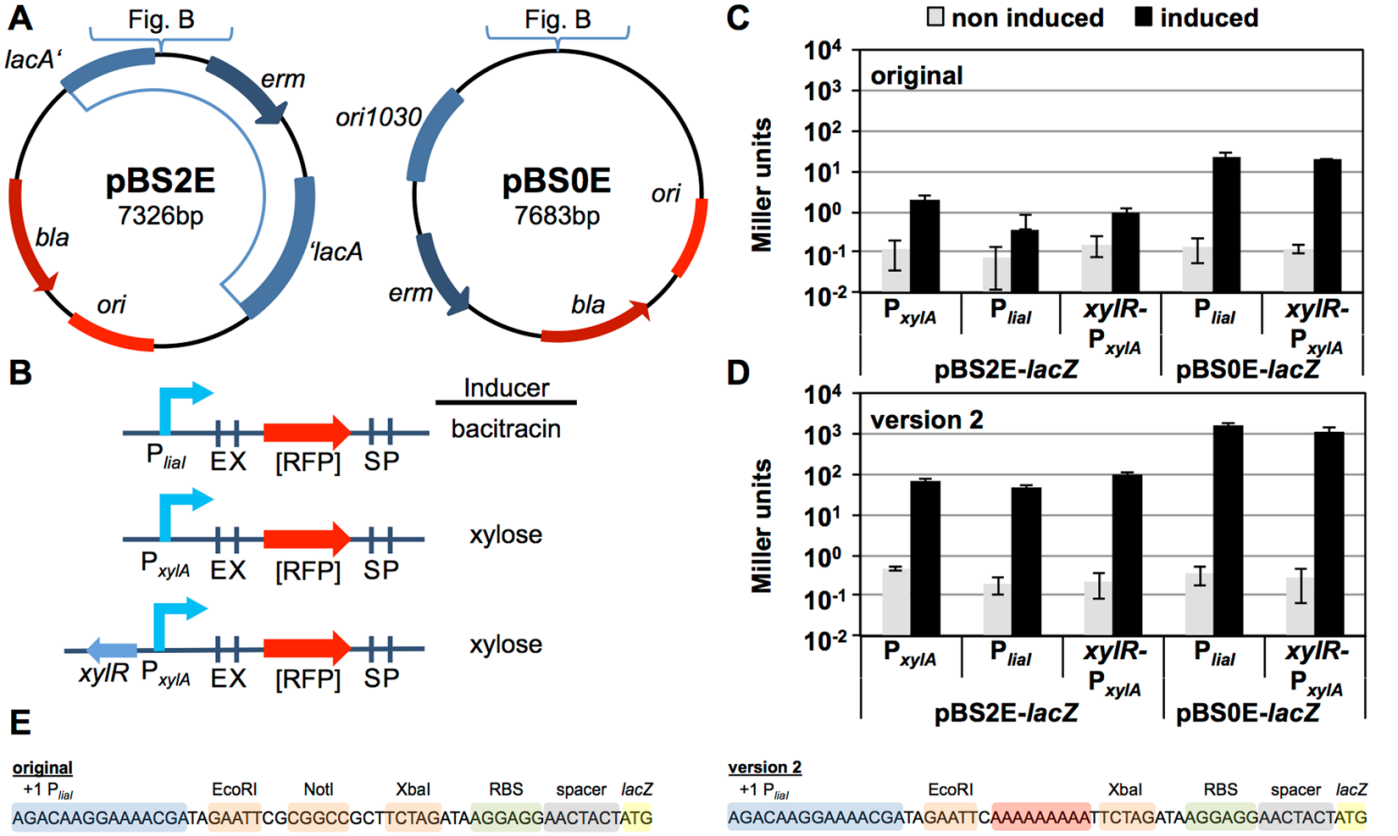
Layout and evaluation of the *Bacillus* BioBrick Box 2.0 expression vectors. (**A**) Vector maps of the original backbones used to generate the expression vectors, pBS2E integrating into the genomic *B. subtilis lacA* locus (hence, it is a single copy version) and pBS0E as a multi copy number version. Both vectors confer resistance against MLS (mediated by *erm*). For the remaining features see legend of Fig. 1. (**B**) Schematic outline of the MCS including one of the three inducible promoters (P_*liaI*_, P_*xylA*_, or *xylR* with P_*xylA*_) and their inducing compounds. (**C**) Observed Miller units of the β-galactosidase assay with strains harboring the original expression vectors and *lacZ* as reporter gene. Gray and black bars represent non-induced and fully induced samples, respectively. Strains TMB3132, TMB3128, TMB3246, TMB3133 and TMB3245 were grown in MCSE medium at 37°C until mid-exponential growth phase, induced with either 30 µg ml^−1^ bacitracin or 0.5% xylose, and harvested 30 min or 60 min after induction, respectively. (**D**) β-galactosidase activities derived from strains harboring the second version of expression vectors (TMB3535 to TMB3537 and TMB3542, TMB3543), using experimental conditions as described in C. (**E**) DNA sequence of the BioBrick prefix for the original expression vectors and the version 2. The *liaI* promoter is depicted as example of the upstream promoter. (**C**,**D**) Show mean values and standard deviations of at least three biological replicates.

Perhaps the most common composite *part* using the BioBrick standard, is the fusion of a promoter with a gene of interest to mediate expression of the latter, e.g. for protein (over)production. For controlled expression, inducible promoters are usually preferred to allow a tight regulation of output in amount and time. The BioBrick 3A (three antibiotic) assembly allows the direct combination of e.g. promoters and genes into an empty vector backbone, hence generating an expression construct in a single reaction^24^. Nevertheless designated expression vectors are desirable to ensure reproducibility. We therefore decided to provide such vectors that are already equipped with suitable, well-described inducible promoters upstream of a BioBrick-compatible MCS. This allows inserting the genes of interest directly into the target vector, without the need of a prior assembly step. These expression vectors are provided both as single-copy, integrative versions as well as on multi-copy replicative backbones by re-designing the empty vectors pBS2E and pBS0E (Fig. 2A). The first step was the insertion of one out of three different inducible promoters: P_*liaI*_, P_*xlyA*_ or a version of P_*xylA*_ in combination with its repressor *xylR* (*xylR-*P_*xylA*_), upstream of the BioBrick prefix on each vector (Fig. 2B). The *xylA* promoter responds to xylose by de-repressing the native regulator XylR^25–27^. For the backbone pBS0E, only *xylR-*P_*xylA*_ was chosen, since we observed that the *xylA* promoter in the absence of an additional vector-based copy of the corresponding repressor XylR showed a high basal promoter activity on replicative vectors (data not shown).

The *liaI* promoter is regulated by the LiaRS two-component system and is strongly induced by sub lethal concentrations of the peptide antibiotic bacitracin^25,28^. Because of its beneficial features – a very low basal activity and a concentration-dependent induction coupled to a very high expression dynamics (1,000-fold over non induced basal activity) – it has already been developed into the LIKE protein-expression system^22,29^. To evaluate our new expression vectors, we chose the reporter gene *lacZ* and performed β-galactosidase assays. Surprisingly, the initial constructs showed very low or even no detectable activation in the presence of the inducers: about 20 Miller units for the pBS0E-derived constructs versus approx. 1 Miller unit in case of pBS2E backbones, respectively (Fig. 2C). These results were in stark contrast to the well-established behavior of both promoters. The sequences of both the promoter and the coding sequence of the reporter gene are fixed and have been shown to work well together in previous constructs, including a combination of the previously developed BioBricks of the respective promoters and the reporter gene (data not shown). This only left the region that was generated by inserting the promoters upstream of the BioBrick prefix as a likely source of this aberrant behavior (for detailed description on the development of the expression vectors see Supplemental text T1). *In silico* analysis lead to the conclusion that the GC-rich NotI restriction site interfered with the ribosome binding site (RBS) of our reporter gene by forming a secondary structure on mRNA levels. This could explain the minimal expression and thus the low measured Miller units. To test this hypothesis we exchanged the NotI site with a poly-A stretch of the same length (Fig. 2E). Evaluation of the newly generated expression vectors demonstrated a full recovery of functionality when compared to the standard BioBrick transcriptional fusion method and resulted upon induction of approximately 100 Miller units for the single copy and up to 1000 Miller units for the multi copy number backbones, respectively (Figs 2D and S4A,C). Additionally, we also tested a BioBrick prefix with only on nucleotide change (Figure S4C) aiming at preserving the NotI restriction enzyme site but trying to decrease the formation of secondary structures. Evaluation of the version 1 expression vectors, showed again very low measured outputs upon induction of only 10 miller units for pBS2E backbones and 20 or 100 Miller units for the P_*liaI*_ and the *xylR*-P_*xylA*_ on the replicative vector, respectively. These differences in expression levels between integrative and replicative vectors is in good agreement with the results obtained for the LIKE expressions system^22,29^. In general, a replicative expression vector usually leads to higher protein production levels due to its higher copy number, but requires maintaining a constant selective pressure to prevent loss. Integrative expression vectors, while leading to lower production levels, are stably maintained even in the absence of antibiotic selection^22,29^.

In our vector suite, we provide functional backbones to (over)express a gene of interest under the control of different inducible promoters. Nevertheless, we strongly recommend to perform bioinformatics analyses, such as predictions of secondary structure formation and translation initiation rates. Moreover, it is important to consider the genetic context of the desired assembly before choosing our expression vectors^30–32^.

### A promoter-screening system based on the highly correlated *cat-lux* reporter pair

**Figure 3:**
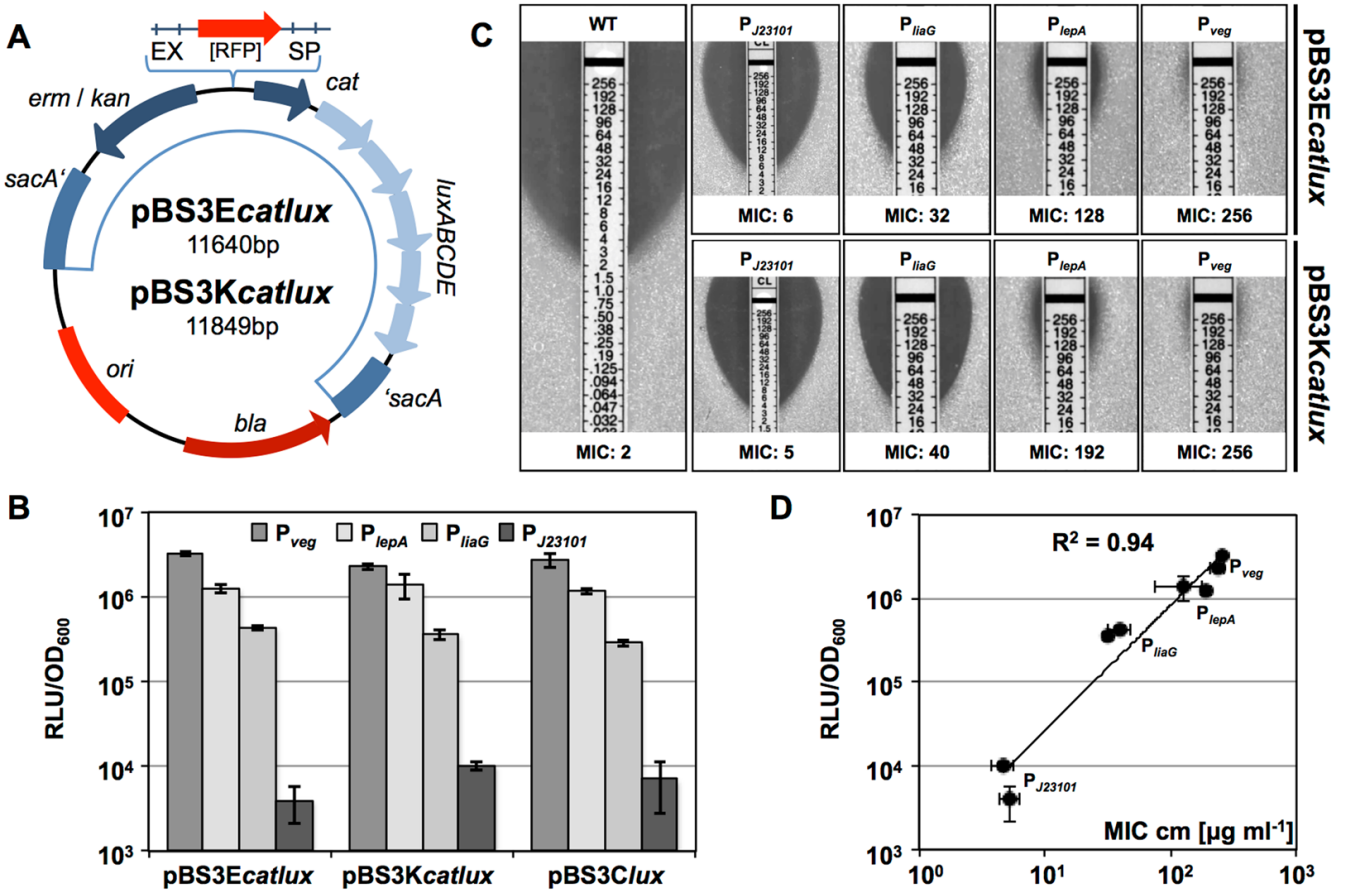
Evaluation of *cat-lux* vectors for promoter screening. (**A**) Vector map of *cat-lux* vectors, which confer resistance either against MLS (mediated by *erm*, pBS3E*catlux*) or kanamycin (mediated by *kan*, pBS3K*catlux*). Both vectors harbor the *cat-lux* reporter system and integrate into the *sacA* locus. For the remaining vector features see description of Fig. 1. (**B**,**C**,**D**) Four constitutive promoters of decreasing strengths (P_*veg*_, P_*lepA*_, P_*liaG*_, and P_*J23101*_) were cloned into both *cat-lux* vectors as well as pBS3C*lux*, which lacks the *cat* gene upstream of the *luxABCDE* cassette. Strains carrying the integrated plasmids (TMB3196-TMB3199 for pBS3E*catlux*, TMB3192-TMB3195 and TMB2940, TMB3090, TMB3212 and TMB3213, respectively) were analyzed for luminescence output (**B**) MIC of chloramphenicol (**C**) and the correlation of both values (**D**). **(B)** Relative luminescence values normalized to cell density (OD_600_) in mid-exponential growth phase were measured in a microtiter plate reader for growth in MCSE medium at 37 °C. (**C**) Determination of chloramphenicol MIC with E-tests® (bioMérieux) on solid MH medium in strains harboring the *cat-lux* vectors with one of the four different constitutive promoters and the wild-type. Representative pictures and mean MIC values are shown. (**D**) Correlation between observed MIC values and relative luminescence as shown in **B** and **C**, both on logarithmic scale. (**B**,**C**,**D**) Show mean values and (**B**,**D**) standard deviations of at least three biological replicates.

With the construction of pBS3K*catlux* and pBS3E*catlux* (Fig. 3A), we developed a new vector-based method to screen promoter libraries. Its logic is derived from the previously described *kan*-*lacZ* system, but offers improved features^33^. Both systems allow a two-step screen. In a first step, the antibiotic concentration is used as a cut-off selection criterion to define the minimal promoter activity desired. In a second step, individual candidate strains are then evaluated quantitatively and in detail by performing luciferase assays. The *lux*-reporter provides a higher dynamic range (signal over background ratio), and increased temporal resolution compared to the dual-reporter systems available so far^34–36^.

We chose four well-studied constitutive promoters (P_*veg*_, P_*lepA*_, P_*liaG*_ and P_*J23101*_) to calibrate our system^19^. First, we checked if the *cat* gene upstream of the *luxABCDE* operon has an influence on the luciferase readout. We compared the four promoters in the promoter-screening vectors with the corresponding constructs in their original *lux*-reporter vectors that lack the *cat* gene. The four promoters behaved almost identical, irrespective of the presence or absence of the *cat* gene (Fig. 3B). No loss in sensitivity of the readout was observed, and even the weakest promoter, P_*J23101*_, was still capable of driving the expression of the *luxABCDE* operon despite the additional presence of the *cat* gene. Only in stationary phase did we observe minor differences between the backbones (Supplemental Figure S3), which have no impact on the functionality.

Since the *cat* gene serves as a co-selection marker to evaluate the promoters, we expected that the chloramphenicol resistance scales with the promoter strength^37^. Next, we therefore analyzed the correlation between promoter strength (as monitored by luminescence) and resistance by determining the minimal inhibitory concentration (MIC) for chloramphenicol (Fig. 3C). The observed MIC values (Fig. 3C) ranged from 2 µg ml^−1^ (wild type) to 256 µg ml^−1^ in a strain with the strongest promoter, P_*veg*_, inserted into our *cat-lux* system. The observed resistance did reflect the order of promoter strengths very well. In fact, we could demonstrate a high degree of correlation (R^2^ value of 0.94) between the two outputs of the vectors, chloramphenicol resistance and luciferase activity (Fig. 3D). Our *cat-lux* vectors are therefore very well suited to isolate and characterize promoters of a desired strength, due to the very good correlation between the two read-outs of the two-step selection procedure outlined.

### Evaluation of RBS libraries using the *lacZ* or *lux*-reporter

In addition to selecting promoters according to their strength, SynBio projects often require to fine tune protein production at the level of translation initiation^38^. This step involves the recognition of specific messenger-RNA elements near the start region of the coding sequence by the 30S ribosomal subunit^39^. At this ribosome binding site (RBS), the 16S rRNA pairs with the Shine-Dalgarno (SD) sequence on the mRNA^40,41^. Statistical analysis revealed that most SD-sequences in *B. subtilis* are very strong and close to the consensus sequence AA AGG AGG^42^.

**Figure 4:**
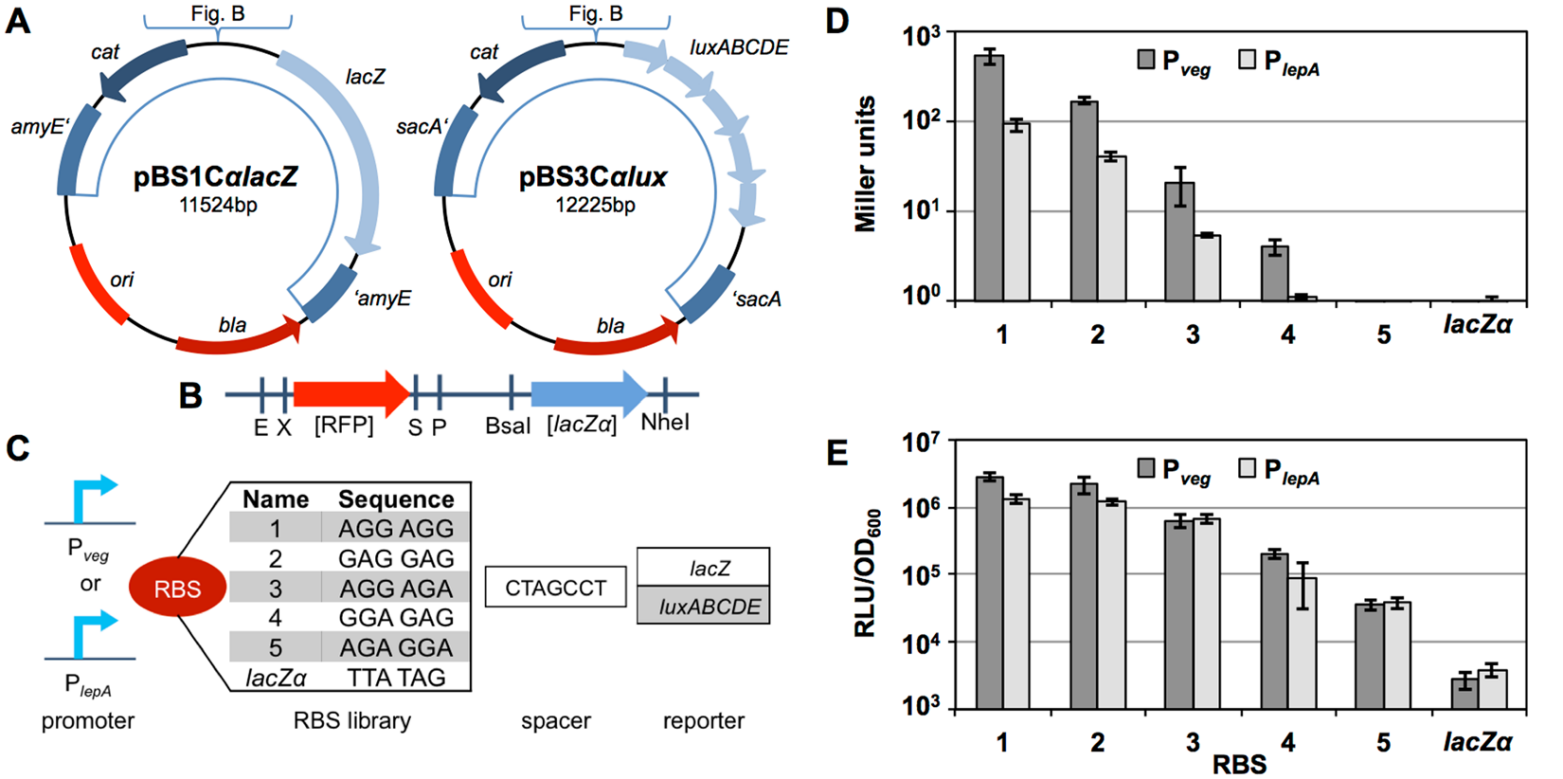
Evaluation and layout of the *Bacillus* BioBrick Box 2.0 RBS evaluation vectors. (**A**) Vector maps of the two RBS evaluation vectors differing in the reporter (light blue) of either *lacZ* or the *luxABCDE* operon. For the remaining features, see legend of Fig. 1. (**B**) Detailed scheme of the MCS comprising the sites for the insertion of a promoter and the RBS sequence upstream of the respective reporter. The promoter of choice replaces the RFP cassette and the RBS replaces the *lacZα* fragment, so both cloning steps can be independently selected for by red/white or blue/white screening, respectively. **(C)** Schematic representation of the design used for evaluation. Two constitutive promoters were chosen (P_*veg*_ and P_*lepA*_) both followed by one out of five different RBS sequences. The spacer sequence accounts for both vector types followed by the specific reporter on each vector. **(D)** Observed β-galactosidase activities of strains harboring the pBS1C*αlacZ* RBS evaluation vector with one of the two constitutive promoters P_*veg*_ (dark gray bars) or P_*lepA*_ (light gray) and the different RBS sequences (1–5) or no inserted RBS (*lacZα*) (P_*veg*_: TMB3182-TMB3186 and TMB2924, P_*lepA*_: TMB3172-TMB3176 and TMB2913). Experimental conditions are described in Fig. 2C. (**E**) Relative luminescence values divided by OD_600_ of strains harboring the pBS3C*αlux* RBS evaluation vector with one of the two constitutive promoters P_*veg*_ (dark gray bars) or P_*lepA*_ (light gray) and the different RBS sequences (1–5) or no inserted RBS (*lacZα*) (P_*veg*_: TMB3162-TMB3166 and TMB2762; P_*lepA*_: TMB3152-TMB3156 and TMB2761). Experimental conditions are described in Fig. 3B (**D**,**E**) show mean values and standard deviations of at least three biological replicates.

To allow investigating RBS libraries, we designed two reporter vectors (Fig. 4A) in which a *lacZα* fragment was inserted downstream of the MCS to allow replacement of the native RBS of the reporter (Fig. 4B). Using the RFP in the MCS and *lacZα* at the RBS position, our vector system enables a red/blue/white screening to check for the insertion of a promoter into the MCS and a RBS replacing the *lacZα* (Supplemental Figure S5). We implemented both the *luxABCDE* operon and the *lacZ* gene as reporters for characterizing RBS libraries based on their luciferase or β-galactosidase activity. In case of the *luxABCDE* reporter operon, only the RBS of *luxA* is replaceable. The *luxA* gene encodes the α-subunit of the luciferase and thus plays a crucial role for luminescence signal^43^.

We chose five RBS sequences and two different constitutive promoters, P_*veg*_ and P_*lepA*_ to evaluate our two vectors (Fig. 4C). RBS 1 matches the consensus RBS sequence of *B. subtilis*, while all other sequences differ in one or more nucleotides from the consensus sequence (Fig. 4C). Using the RBS calculator from the Salis lab^30,44^, we predicted the expected translation initiation rate of each construct (Supplement Table S3). Not surprisingly, the predictions for each RBS differ between *luxA* and *lacZ*, due to the known context dependence of translation initiation^32^.

The *in vivo* evaluation revealed a gradual decrease in the measured output from RBS 1 to RBS 5 irrespective of the promoter. These results are in good agreement with the predictions mentioned above, especially for the *luxABCDE* operon as reporter gene, which was initially used to generate the RBS library (Fig. 4E and Table S3 top). Not surprisingly, the absolute values differ between the two promoters driving reporter gene expression, especially for *lacZ*. These observations confirm previous results demonstrating that P_*veg*_ is a stronger constitutive promoter than P_*lepA*_.

The β-galactosidase activities ranged from about 500 (RBS1) to 4 (RBS4) Miler units for constructs carrying the P_*veg*_ promoter, while 90 to 1 Miller units were reached with P_*lepA*_, using the same RBS sequences. No β-galactosidase activity could be detected for RBS5, irrespective of the promoter (Fig. 4D). With the *lux*-reporter, the difference between the two constitutive promoter is less pronounced when compared to *lacZ* (Fig. 4D, E). Again, the *lux* reporter provides a much higher dynamic range and hence significantly improved resolution than *lacZ*. Even the ‘empty’ constructs with *lacZα* inserted instead of an RBS upstream of the reporter gene gave rise to detectable, although very low luciferase activity. This is reasonable since the *lacZα* fragment shows the highest sequence deviation from the consensus RBS sequence of *B. subtilis*. In contrast, no activity was observed for either RBS 5 or *lacZα* was observed in the β-galactosidase assay, even in the presence of the strongest promoter, P_*veg*_ (Fig. 4D, E).

Taken together, our proof of principle study with five RBS of different strengths demonstrates the functionality of our two screening vectors. They allow identifying RBSs differing about two orders of magnitude in strength, thereby enabling a fine-tuning of translation initiation for applied projects. Our vector system should therefore also allow a quick and easy screening of larger RBS libraries to identify RBS of a desired strength even from complex mixtures of sequences.

### Evaluation of fluorescent proteins in *B. subtilis*

Since their discovery, fluorescent proteins (FPs) have become valuable tools for molecular biologists. Applications are as numerous as the available protein variants, ranging from classical reporters for gene expression over to advanced microscopy techniques and genetically encoded biosensors. The proteins themselves are subject to continuous engineering to achieve additional color variants, improved brightness, and stability^45,46^.

**Figure 5:**
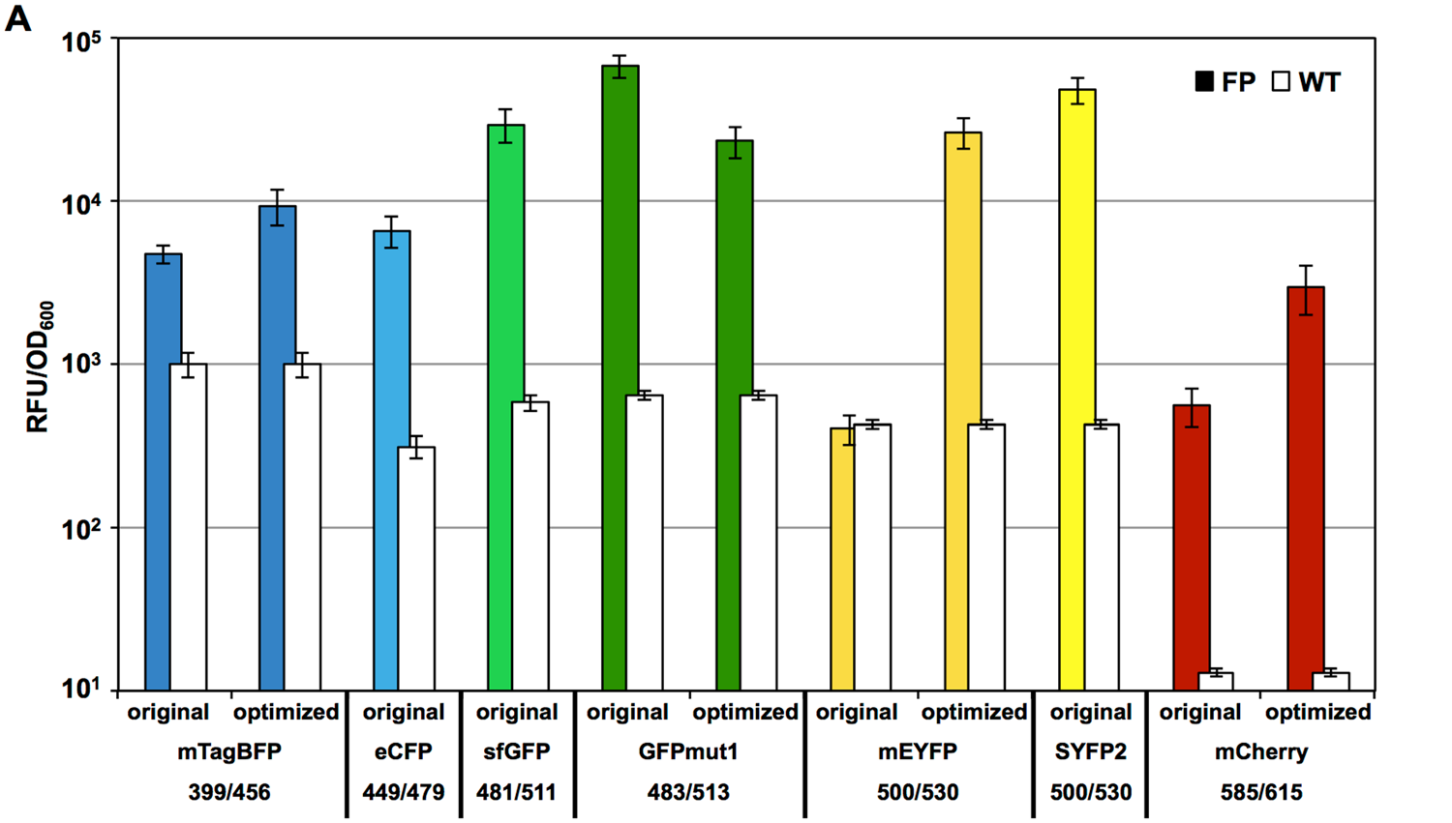
Endpoint measurements of the fluorescent proteins contained in the *Bacillus* BioBrick Box 2.0. Relative fluorescent units normalized by optical density (OD_600_) are shown for cells with fully induced FP expression (colored bars), compared to the autofluorescence of *B. subtilis* wild type cells (white bars). Depending on the FP, original as well as optimized versions were tested. The excitation and emission wavelengths are depicted for each FP. Bar graphs represent mean values and standard deviations of at least three biological replicates. The corresponding strains (TMB3909-TMB3921) carry the genes encoding the respective FP (RFC 10) under control of the bacitracin-inducible promoter P_*liaI*_. For evaluation of the spectra (Figure S6) and endpoint measurements, cells were grown to exponential phase and expression of the FPs was induced by addition of bacitracin (final concentration 30 µg ml^−1^) for 75 min to allow proper folding of the FPs. After induction, cells were harvested and washed with PBS and all measurements were performed in a final volume of 200 µl in 96 well plates using a Synergy™ NeoalphaB plate reader.

Here, we evaluated seven different FPs ranging from the blue to red spectra and aimed at improving their use in *B. subtilis* by codon optimization (Table 2). All seven FP variants are provided in RFC10 standard, thereby allowing transcriptional fusions. In addition, three of them – sfGFP, SYFP2 and mCherry – are also provided in the RFC25 standard, thereby enabling the convenient construction of translational fusion proteins. All FPs were cloned into the single copy number backbone pBS1C and transcriptionally fused with the inducible P_*liaI*_ promoter via standard 3A BioBrick assembly^8^. Endpoint measurements of the corresponding *B. subtilis* strains are shown in Fig. 5.

**Table 2:** Collection of available fluorescent proteins.

Protein	Ex [nm]^1^	Em [nm]^1^	Codon usage (original/adapted)	Max fluorescence [RFU/OD_600_]^2^ (original/adapted)	Standard	Ref.
mTagBFP	401	455	*E. coli * **/ ** *B. subtilis*	4,700 **/ **9,300	RFC10	60
eCFP	451	477	*B. subtilis*	6,500	RFC10	61
sfGFP	486	511	*S. pneumoniae*	29,300	RFC10 **/ **RFC25	62
mGFPmut1	485	510	*A. victoria * **/ ** *B. subtilis*	67,700 **/ **23,500	RFC10	46
mEYFP	512	529	*E. coli * **/ ** *B. subtilis*	400 **/ **26,400	RFC10	61
SYFP2	514	530	*E. coli*	48,300	RFC10 **/ **RFC25	63
mCherry	585	612	*E. coli * **/ ** *B. subtilis*	600 **/ **3,000	RFC10 **/ **RFC25	64

^1^Acutal wavelengths observed from the spectra measurements (Figure S6) determining the maxima of excitation and emission for a FP (in case of several codon usage versions, *B. subtilis* codon usage values are depicted).

^2^Depicted values are rounded values.

First, we determined the excitation and emission maxima for each FP (Tables 2, S4). For this, we chose fixed wavelengths and varied illumination in 1 nm steps over a range of 110–180 nm to record both spectra (Supplemental Figure S6 and Table S4). The determined maxima were then used to perform the endpoint measurements (Table 2 and Fig. 5, colored bars). If the respective excitation/emission maxima were too close to exclude blead through, the detected emission wavelength was increased until the minimal excitation/emission gap was reached. For each FP, we also tracked the auto fluorescence of the wild type (Fig. 5, white bars).

All FPs, with the exception of the original mEYFP, show a fluorescence signal of at least one order of magnitude above the background fluorescence of the wild type in a single copy number set-up, based on their integration into the *B. subtilis* chromosome. With over two orders of magnitude, the signal over background ratio is especially large for mCherry. Between 400 and 500 nm excitation wavelength (all FPs except mCherry), the observed autofluorescence of the wild type ranged between 500 and 1,000 RFU/OD_600_. In contrast, the wild type strain has a very low autofluorescence of only 13 RFU/OD_600_ at the wavelength required to excite mCherry (Fig. 5).

mTagBFP gives rise to 4,700 and 10,000 RFU/OD_600_ in its original and optimized version, respectively. GFP and eCFP both show a strong signal of up to two orders of magnitude above background autofluorescence. The observed relative fluorescence of sfGFP, codon optimized for *Streptococcus pneumoniae*, which was previously reported to work best in *B. subtilis*, was notably lower compared to the original GFPmut1^47^. While the original version of the yellow mEYFP showed no signal over wild-type background, codon optimization resulted in a mEYFP signal of about 30-fold over the autofluorescence. Comparing the optimized mEYFP with the original SYFP2 shows that SYFP2 has an even higher relative fluorescence in *B. subtilis* (about 48,000 RFU/OD_600_). The original mCherry gives rise to 560 RFU/OD_600_ while the optimized version results in an over five-fold increased maximum fluorescence output of about 3,000 RFU/OD_600_, that is, more than 200-fold above the autofluorescence. It is therefore a particularly well-suited FP, despite its moderate absolute fluorescence output (Table 2 and Fig. 5).

The comparison between original FPs and their codon-optimized derivatives revealed that an increase of observed fluorescence could be achieved for all but one FP (Table 2 and Fig. 5). Especially for the optimized mEYFP, a 64-fold change compared to the original was achieved. In case of the red fluorescent protein mCherry, the optimized version had a five-fold higher intensity. The only exception was the optimized version of GFPmut1, which showed a slight decrease in RFU/OD_600_ when compared to the original protein.

Taken together, this set of FPs, which covers the whole spectrum of visible light, provides powerful reporter genes for the work in *B. subtilis*. For dual labeling experiments within a single cell, both the YFP/CFP and the GFP/mCherry pairs are useful.

## Conclusion

This study provides a significant extension of the previously described *Bacillus* BioBrick Box^19^. It offers a larger range of empty integrative vectors, a set of expression vectors, as well as screening tools for isolating and characterizing promoters and ribosome binding sites of a desired strength. In addition, a set of FPs was codon-optimized evaluated for its use in the Gram-positive model organism *B. subtilis*. All vectors and parts are comprehensively evaluated to demonstrate their performance in this organism.

For the empty backbones with new combinations of resistance markers, we demonstrated their functionality in β-galactosidase and luciferase assays. A comparison of the newly generated plasmids with the original backbones showed no difference, highlighting their robustness, irrespective of the antibiotic resistance cassettes.

With the constructions of our expression vectors, harboring one out of three inducible promoters upstream of the MCS, we provide a set of useful vectors to conveniently overexpress proteins of interest in *B. subtilis* that have been cloned in the RFC10 standard. Based on inserting the *lacZ* gene and performing β-galactosidase assays, the replicative expression vectors result in an about 15–20-fold higher protein yield compared to the single copy integrative vectors.

Further, we constructed and evaluated a vector-based method to screen and quantitatively evaluate promoter libraries for candidates of interest using a two reporter-system: the *cat* gene and the *luxABCDE* operon. As a final observation to this method, we could demonstrate a high correlation between the mentioned reports allowing to very quickly screen a large repertoire of promoters for candidates of a desired strength by simply setting the chloramphenicol concentration as threshold.

In the course of evaluating our second toolbox, we identified a number of potential drawbacks to the BioBrick standard, despite its undisputed usefulness in routine lab cloning. First, the RFC10 prefix lead to weak expression in our first design of expression vectors, most likely due to the formation of secondary structures (Figs 3 and S4). Second, the RFC25 prefix – while enabling translational fusions – lowered the fluorescence signal of all three FPs tested in comparison to transcriptional fusions of the RFC10 – versions (Supplemental Figure S7). Despite these drawbacks of the BioBrick standard, we believe that this carefully evaluated *Bacillus* BioBrick Box 2.0 is a powerful expansion of the first *Bacillus* BioBrick Box, which has found widespread use both in the iGEM competition and beyond.

Development of toolboxes is crucial for standardized work and reproducibility, especially in the field of SynBio. Accordingly, it has gained considerable interest and a number of recent studies also provide well-evaluated tools for SynBio applications in *B. subtilis* in addition to the second *Bacillus* BioBrick box described in this article. A recent study described a set of promoters, ribosome binding sites and proteolytic tags provides a vector for carefully tuning protein expression in *B. subtilis*, both at the transcriptional and translational level^48^. Another study specifically focused on the development of a method for transferring very large fragments into *B. subtilis* ^49^. Additionally, our group most recently introduced the Standard European Vector Architecture (SEVA) to the *Bacillus* community. The resulting SEVA-sibling collection provides a modular vector framework that can be easily personalized to any integration site and allows a broad range of application even beyond the *B. subtilis* reference laboratory wild type strain 168 (Radeck *et al*. in press). In addition to this valuable efforts, we now provide a powerful and stand-alone collection of standardized genetic *parts*, all adhering to the BioBrick cloning standard (Table 1). Together with the first installment of the *Bacillus BioBrick box* ^19^, this extensive toolbox now allows to genetically modify *B. subtilis* for performing a large variety of different experiments, based on a highly compatible set of vectors and well-evaluated *part*s. We therefore hope that our new tools will prove to be just as useful for *Bacillus* researchers around the world.

## Material and Methods

### Reagents

Chemicals used in this study were obtained from *Thermo Scientific* (Waltham, MA, USA), *Carl Roth GmbH & Co. KG* (Karlsruhe, Germany) or *Sigma-Adrich* (St. Louis, MO, USA). All enzymes (restriction endonucleases, ligases and polymerases for PCR) originated from *New England Biolabs* (Ipswich, MA, USA) and general cloning procedures were performed according to the recommended protocols. PCR purifications and plasmid preparations were undertaken using the corresponding kits from *Süd-Laborbedarf GmbH* (Gauting, Germany).

### DNA manipulation and plasmid construction

General cloning procedure followed the protocols described in Radeck *et al*.^19^, with the exception of using the Q5 polymerase for PCR amplification. All strains and primers generated in this study are listed in Tables S1 and S2, respectively.

### Vector construction

All parts (Table 1) were verified by sequencing and Genbank-files are provided in Supplemental Material (Additional File 1).

Construction of the reporter vectors pBS3K*lux* and pBS3E*lux* was performed by PCR amplification of pBS3C*lux* ^19^ with primers TM4185 and TM4186 to generate a *cat*-free fragment. The new *B. subtilis* antibiotic resistance cassettes *kan* and *erm*, were generated by using pDG780^50^ and pDG647^50^ as templates with primers TM4187/TM4188 and TM4302/TM4303, respectively. All components were digested with ApaI and NheI followed by a ligation reaction.

To construct pBS1E and pBS1K, the template pBS1C^19^ was amplified with the primers TM4304 and TM4305, resulting in a *cat-*free backbone. The *erm* and *kan* antibiotic resistance cassettes were amplified and ligated into the backbone as described above. The replicative vector pBS0E derives from pGP380^21^. In order to fulfill the requirements of the RFC10 BioBrick standard, one SpeI restriction site was removed by primer mutagenesis (TM3168 and TM3169). The RFP cassette originating from the iGEM backbone pSB1C3F^51^ was inserted into the multiple cloning site (MCS) via EcoRI and PstI.

The promoter evaluation vectors pBS3K*catlux* and pBS3E*catlux* derive from the pBS3K*lux* and pBS3E*lux* vectors, respectively. Both were digested with PstI and SalI, thereby opening the vectors between the MCS and the *luxA* gene. The *cat* gene (including its native RBS) was PCR-amplified with primers TM4317 and TM4318, using pBS3C*lux* as the template. The PCR product was then digested with PstI and SalI and ligated into both vectors.

The expression vectors harboring the inducible promoters P_*liaI*_, P_*xylA*_ and *xylR*-P_*xylA*_ upstream of the MCS, were generated from plasmids 1011^19^, 1076^19^ or 1049^19^ as sources for the promoters. PCR amplification was performed using the primers TM4069/TM4070, TM4067/TM4068 and TM4071/TM4072, respectively. The PCR products were then digested with BsaI and EcoRI, leaving EcoRI specific base pair overhangs on both sides. The backbones pBS2E and pBS0E were linearized with EcoRI and ligated with each of the three promoters. Sequencing reactions confirmed all five resulting vectors. The β-galactosidase assay was chosen for evaluation. Towards that goal, the *lacZ* gene from pSB1C3-*lacZ* (TME1020^19^) was cloned in all expression vectors via EcoRI and PstI. To create the expression vectors version 1 and 2, primers TM4821 and TM4822 as well as TM4823 together with TM4824 were annealed and ligated into EcoRI and XbaI cut original expression vectors.

Construction of the RBS evaluation vector pBS3C*αlux* was achieved by PCR amplification of pBS3C*lux* as template with the primers TM3159 and TM3160. The PCR product of *lacZα* was amplified from the template BBa_I732902^52^ using the primers TM3854 and TM3855. Both PCR fragments were digested with BsaI and NheI followed by a ligation step, during which the RBS of the *luxA* gene was eliminated.

The second RBS evaluation vector, pBS1C*αlacZ*, was generated by site-directed mutagenesis of the pBS1C*lacZ* ^19^ with the primers TM3163 and TM3164 to remove one BsaI restriction enzyme site. The resulting vector was checked via test digest with BsaI. This pBS1C*lacZ*(-BsaI) intermediate was amplified with TM4250 to give rise to a NheI restriction enzyme site. The MCS and *lacZα* part from the pBS3C*αlux* was ligated into pBS1ClacZ(−BsaI + NheI) via EcoRI and NheI.

### The RBS library

For the design of the RBS library, the Salis lab *RBS calculator* ^30,44^ was used and five RBS sequences were selected that were predicted to have a range of translation initiation rate of over two orders of magnitude. The specific sequences used for the prediction are provided in Table S3. The library was ordered as primers designed to anneal with overhangs compatible for insertion into pBS3C*αlux* and pBS1C*αlacZ* (TM4325 till TM4334), two primers forming one RBS in the library. They were incubated at 95°C for 5 min and cooled down to RT. The annealed primer pairs were then ligated into both vectors via BsaI and NheI, replacing *lacZα*. P_*lepA*_ and P_*veg*_ were incorporated into the MCS via EcoRI and PstI.

### Codon optimization of the fluorescent proteins

The Codon Adaptation Index (CAI) proposed by Sharp and Li (1987)^53^ was used to quantify the adaption of FP-encoding genes to the *B. subtilis* codon usage. For CAI_BSU_, codon frequencies were compared to those obtained from the Kazusa codon usage database, which is based on the analysis of all *B. subtilis* genes regardless of their expression levels^54^. The CAI was calculated using a customized version of the AutoAnnotator created by the iGEM Team TU-Munich (2013)^55^. The FP-encoding genes were synthesized by GeneArt^®^ and marked by addition of “BSU” to the protein name (except of GFPmut1, for which the optimized variant is marked by the addition of “LT”, because the LifeTech® codon adaptation algorithm was used). For sfGFP we used the *Streptococcus pneumoniae* codon adapted version, which was previously described to work best for *B. subtilis* ^47^.

The FP’s were cloned into the single copy integrative vector pBS1C and transcriptionally fused to the promoter P_*liaI*_.

### Bacterial growth conditions and physiological methods

All strains (Table S1) were grown at 37 °C, with aeration (200 rpm agitation) in one of the following media: (i) Lysogeny broth (LB medium) [0.5% (w/v) yeast extract, 1% (w/v) tryptone, 1% (w/v) sodium chloride]; (ii) MNGE medium^56^; (iii) MCSE medium^19^; (iv) Müller Hinton broth (MH-medium) [2.1% (w/v) mueller-hinton broth; Carl Roth]. For solid agar plates 1.5% (w/v) agar-agar (Carl Roth) was added or 0.75% (w/v) for soft agar plates, respectively. *E. coli* cells harboring a plasmid were selected on ampicillin (100 μg ml^−1^) or chloramphenicol (35 μg ml^−1^). *B. subtilis* cells carrying a resistance marker were selected using chloramphenicol (5 μg ml^−1^), kanamycin (10 μg ml^−1^) or erythromycin combined with lincomycin (1 μg ml^−1^, 25 μg ml^−1^) for MLS. Transformation of *E. coli* and *B. subtilis* was performed as described previously^56,57^. Determination of the minimal inhibitory concentration (MIC assays) of *B. subtilis* against chloramphenicol was performed using eTests (bioMerieux, range 0.0x-256 mg ml^−1^) according to the procedure described in Radeck *et al*.^58^.

### Reporter gene assays

The luciferase assay was performed as described previously^19^, with the following changes: *B. subtilis* cells were grown in MCSE media and analyzed using the Synergy™ NeoalphaB plate reader (BioTek, Winooski, VT, USA). OD_600_ as well as luminesce was monitored every 5 minutes for at least 15 hours.

The β-galactosidase assay was basically performed as described previously^19^, but using cells grown in MCSE media. In brief, overnight cultures were inoculated in MCSE with the correct antibiotic. The cultures were inoculated 1:200 in fresh MCSE w/o antibiotic on the following day and grown to an OD_600_ of 0.4–0.9. After that 2 ml of each culture was harvested by centrifugation (13000 rpm, 10 min, 4°C) and resuspended in 1 ml working buffer [Z-buffer (60 mM disodium phosphate ×2 H_2_O, 40 mM monosodium phosphate × H_2_O, 10 mM potassium chloride, 1 mM magnesium sulfate × 7 H_2_O), 20 mM β-mercaptoethanol]. After that cells were diluted with working buffer until an OD_600_ of 0.2–0.8 in a final volume of 800 μl was reached, then 10 μl lysozyme [15 mg ml^-1^ in Z-buffer] was added followed by an incubation at 37°C for one hour. After the incubation, 150 μl ONPG [2-nitrophenyl-β-D-galactopyranoside] was added and the time until the sample turns yellow was measured. The reaction was stopped by applying 400 μl 1 M sodium carbonate. Finally measurements of OD_420_ as well as OD_550_ were performed, while using a blank containing all components mentioned above, excluding cells. The β-galactosidase activity was normalized to the cell density using the equation described by Miller, 1972^59^.

### Evaluation of the fluorescent proteins

For quantification of fluorescence spectra and intensities, *B. subtilis* cells were analyzed using the Synergy™ NeoalphaB plate reader (BioTek, Winooski, VT, USA) with monochromators for illumination. Day cultures were inoculated 1:50 from fresh overnight cultures, both were grown in LB medium. Cells were induced in exponential growth phase with 30 µg ml^−1^ of bacitracin and 2 ml of cells were harvested 75 minutes after induction to allow proper folding of the FPs. The cells were washed once with 1 ml phosphate buffered saline (PBS), resuspended in 1 ml PBS and 200 μl were filled into each well of a 96-well plate (black, clear bottom; Greiner Bio-One, Frickenhausen, Germany) followed by readings from the top without lid.

To record emission spectra, cells were illuminated at a fixed excitation wavelength and emitted fluorescence was recorded over a range of 110–150 nm in 1 nm steps, see Table S4. For excitation spectra, illumination was varied in 1 nm steps over a range of 110–180 nm and emission was measured at a fixed wavelength. For endpoint quantifications, both excitation and emission wavelengths were fixed in a distance of at least 30 nm, where the emission wavelength was chosen to be at the reported emission maximum, leading to a non-optimal excitation for those FPs where the distance between both maxima was smaller than 30 nm. For all measurements, the gain was fixed to 100. All parameters were kept constant for measuring both codon variants of the same protein as well as for wild type and blank PBS controls. To normalize on fluorescence per cell, sample values were divided by the OD_600_. The recorded spectra (excitation and emission) were normalized to the wild type spectrum of each fluorescent protein. Mean values and standard deviation were determined from at least biological triplicates.

## Electronic supplementary material

Supplementary Information
