# Supplementary material for: The Bacillus BioBrick Box 2.0: expanding the genetic toolbox for the standardized work with Bacillus subtilis
Source: Sci Rep. 2017 Nov 8;7:15058. doi: 10.1038/s41598-017-15107-z (PMC5678133; doi:10.1038/s41598-017-15107-z)
Supplement: Supplementary file 1 — Supplementary Information [file 41598_2017_15107_MOESM1_ESM.pdf]

# The *Bacillus* BioBrick Box 2.0: expanding the genetic toolbox for the standardized work with *Bacillus subtilis*

Popp F Philipp[1], Dotzler Mona[2], Radeck Jara[1,2], Bartels Julia[1,2] and Mascher Thorsten[1*]

[1]Institute of Microbiology, Technische Universität (TU) Dresden, Dresden, Saxony, 01062, Germany

[2]Institute of Microbiology, Ludwig-Maximilians-Universität (LMU) München, Planegg-Martinsried, Bavaria, 82152, Germany

* To whom correspondence should be addressed. Tel: +49 351 463-40420; Fax: +49 351 463-37715; Email: Thorsten.Mascher@tu-dresden.de; Present Address: Thorsten Mascher, Institute for Microbiology, TU Dresden, Dresden, Saxony, 01217, Germany

Philipp F Popp : philipp.popp@tu-dresden.de

Mona Dotzler: mona.dotzler@campus.lmu.de

Jara Radeck: jara.radeck@tu-dresden.de

Julia Bartels: Julia.bartels@tu-dresden.de

Thorsten Mascher: Thorsten.mascher@tu-dresden.de

## Additional File 1

Table of content:

Table S1: Strains generated in this study

Table S2: Primers used in this study

Table S3: Sequences used for the Salis RBS calculator

Table S4: Wavelengths used for plate reader measurements in nm

Figure S1: Evaluation of the new *lux*-reporter vectors

Figure S2: Evaluation of the new empty *amyE* integrative vectors

Figure S3: Evaluation of the *catlux* promoter screening vectors

Figure S4: Assembly layout stages of the *Bacillus* BioBrick Box 2.0 expression vectors

Figure S5: Cloning stages of the RBS exchangeable screening vectors

Figure S6: Excitation and emission spectra of the *Bacillus* BioBrick Box 2.0 fluorescent proteins

Figure S7: Fold change in measured fluorescence of different RFC standards

Supplement text 1: Comment on the design aspects regarding the *Bacillus* BioBrick Box 2.0 expression vectors

## Table S1: Strains generated in this study

| **Name** | **Description and comment[1]** | **Source** |
|---|---|---|
| ***B. subtilis*** | | |
| W168 | *trpC2* | lab strain |
| TMB2940 | W168 *sacA*::pBS3C-$P_{lepA}$-*lux* | This study |
| TMB3088 | W168 *sacA*::pBS3E-$P_{lepA}$-*lux* | This study |
| TMB3089 | W168 *sacA*::pBS3K-$P_{lepA}$-*lux* | This study |
| TMB3090 | W168 *sacA*::pBS3C-$P_{veg}$-*lux* | This study |
| TMB3091 | W168 *sacA*::pBS3E-$P_{veg}$-*lux* | This study |
| TMB3092 | W168 *sacA*::pBS3K-$P_{veg}$-*lux* | This study |
| TMB3204 | W168 *amyE*::pBS1C-RFP | This study |
| TMB3205 | W168 *amyE*::pBS1E-RFP | This study |
| TMB3544 | W168 *amyE*::pBS1K-RFP | This study |
| TMB3125 | W168 *amyE*::pBS1C-$P_{veg}$-*lacZ* | This study |
| TMB3126 | W168 *amyE*::pBS1E-$P_{veg}$-*lacZ* | This study |
| TMB3547 | W168 *amyE*::pBS1K-$P_{\mathrm{veg}}$-*lacZ* | This study |
| TMB3497 | W168 *amyE*::pBS2E$P_{xylA}$_*lacZ* (3A) | This study |
| TMB3498 | W168 *lacA*::pBS2E$P_{liaI}$_*lacZ* (3A) | This study |
| TMB3499 | W168 *lacA*::pBS2E*xylR*$P_{xylA}$_*lacZ* (3A) | This study |
| TMB3501 | W168 pBS0E$P_{liaI}$_*lacZ* (3A) | This study |
| TMB3502 | W168 pBS0E*xylR*$P_{xylA}$_*lacZ* (3A) | This study |
| TMB3132 | W168 *lacA*::pBS2E$P_{xylA}$-*lacZ* (EV) | This study |
| TMB3128 | W168 *lacA*::pBS2E$P_{liaI}$-*lacZ* (EV) | This study |
| TMB3246 | W168 *lacA*::pBS2E*xylR*$P_{xylA}$-*lacZ* (EV) | This study |
| TMB3133 | W168 pBS0E$P_{liaI}$-*lacZ* (EV) | This study |
| TMB3245 | W168 pBS0E*xylR*$P_{xylA}$-*lacZ* (EV) | This study |
| TMB3532 | W168 *lacA*::pBS2E$P_{xylA}$-*lacZ* (Version 1) | This study |
| TMB3533 | W168 *lacA*::pBS2E$P_{liaI}$-*lacZ* (Version 1) | This study |
| TMB3534 | W168 *lacA*::pBS2E*xylR*$P_{xylA}$-*lacZ* (Version 1) | This study |
| TMB3539 | W168 pBS0E$P_{liaI}$-*lacZ* (Version 1) | This study |
| TMB3540 | W168 pBS0E*xylR*$P_{xylA}$-*lacZ* (Version 1) | This study |
| TMB3535 | W168 *lacA*::pBS2E$P_{xylA}$-*lacZ* (Version 2) | This study |
| TMB3536 | W168 *lacA*::pBS2E$P_{liaI}$-*lacZ* (Version 2) | This study |
| TMB3537 | W168 *lacA*::pBS2E*xylR*$P_{xylA}$-*lacZ* (Version 2) | This study |
| TMB3542 | W168 pBS0E$P_{liaI}$-*lacZ* (Version 2) | This study |
| TMB3543 | W168 pBS0E*xylR*$P_{xylA}$-*lacZ* (Version 2) | This study |
| TMB2761 | W168 *sacA*::pBS3C-$P_{lepA}$-*lacZα*-*lux* | This study |
| TMB2762 | W168 *sacA*::pBS3C-$P_{veg}$-*lacZα*-*lux* | This study |
| TMB2913 | W168 *sacA*::pBS1C-$P_{lepA}$-*lacZα*-*lacZ* | This study |
| TMB2924 | W168 *sacA*::pBS1C-$P_{veg}$-*lacZα*-*lacZ* | This study |
| TMB3152 | W168 *sacA*::pBS-$P_{lepA}$-RBS1-3C*lux* | This study |
| TMB3153 | W168 *sacA*::pBS-$P_{lepA}$-RBS2-3C*lux* | This study |
| TMB3154 | W168 *sacA*::pBS-$P_{lepA}$-RBS3-3C*lux* | This study |
| TMB3155 | W168 *sacA*::pBS-$P_{lepA}$-RBS4-3C*lux* | This study |
| TMB3156 | W168 *sacA*::pBS-$P_{lepA}$-RBS5-3C*lux* | This study |
| TMB3162 | W168 *sacA*::pBS-$P_{veg}$-RBS1-3C*lux* | This study |
| TMB3163 | W168 *sacA*::pBS-$P_{veg}$-RBS2-3C*lux* | This study |

| | | |
|---|---|---|
| TMB3164 | W168 *sacA*::pBS-P$_{veg}$-RBS3-3C*lux* | This study |
| TMB3165 | W168 *sacA*::pBS-P$_{veg}$-RBS4-3C*lux* | This study |
| TMB3166 | W168 *sacA*::pBS-P$_{veg}$-RBS5-3C*lux* | This study |
| TMB3172 | W168 *amyE*::pBS1C-P$_{lepA}$-RBS1-*lacZ* | This study |
| TMB3173 | W168 *amyE*::pBS1C-P$_{lepA}$-RBS2-*lacZ* | This study |
| TMB3174 | W168 *amyE*::pBS1C-P$_{lepA}$-RBS3-*lacZ* | This study |
| TMB3175 | W168 *amyE*::pBS1C-P$_{lepA}$-RBS4-*lacZ* | This study |
| TMB3176 | W168 *amyE*::pBS1C-P$_{lepA}$-RBS5-*lacZ* | This study |
| TMB3182 | W168 *amyE*::pBS1C-P$_{veg}$-RBS1-*lacZ* | This study |
| TMB3183 | W168 *amyE*::pBS1C-P$_{veg}$-RBS2-*lacZ* | This study |
| TMB3184 | W168 *amyE*::pBS1C-P$_{veg}$-RBS3-*lacZ* | This study |
| TMB3185 | W168 *amyE*::pBS1C-P$_{veg}$-RBS4-*lacZ* | This study |
| TMB3186 | W168 *amyE*::pBS1C-P$_{veg}$-RBS5-*lacZ* | This study |
| TMB3192 | W168 *sacA*::pBS3K-P$_{lepA}$-*catlux* | This study |
| TMB3193 | W168 *sacA*::pBS3K-P$_{veg}$-*catlux* | This study |
| TMB3194 | W168 *sacA*::pBS3K-P$_{liaG-}$*catlux* | This study |
| TMB3195 | W168 *sacA*::pBS3K-P$_{J23101}$-*catlux* | This study |
| TMB3196 | W168 *sacA*::pBS3E-P$_{lepA}$-*catlux* | This study |
| TMB3197 | W168 *sacA*::pBS3E-P$_{veg}$-*catlux* | This study |
| TMB3198 | W168 *sacA*::pBS3E-P$_{liaG-}$*catlux* | This study |
| TMB3199 | W168 *sacA*::pBS3E-P$_{J23101}$-*catlux* | This study |
| TMB3212 | W168 *sacA*::pBS3C-P$_{liaG}$-*lux* | This study |
| TMB3213 | W168 *sacA*::pBS3C-P$_{J23101}$-*lux* | This study |
| TMB3909 | W168 *amyE*::pBS1C-P$_{liaI}$ sfGFP_Spn | This study |
| TMB3910 | W168 *amyE*::pBS1C-P$_{liaI}$ mTagBFP | This study |
| TMB3911 | W168 *amyE*::pBS1C-P$_{liaI}$ mTagBFP_Bsu | This study |
| TMB3912 | W168 *amyE*::pBS1C-P$_{liaI}$ eCFP_Bsu | This study |
| TMB3913 | W168 *amyE*::pBS1C-P$_{liaI}$ mEYFP | This study |
| TMB3914 | W168 *amyE*::pBS1C-P$_{liaI}$ mEYFP_Bsu | This study |
| TMB3915 | W168 *amyE*::pBS1C-P$_{liaI}$ mCherry | This study |
| TMB3916 | W168 *amyE*::pBS1C-P$_{liaI}$ mCherry_Bsu | This study |
| TMB3917 | W168 *amyE*::pBS1C-P$_{liaI}$ mGFPmut1 | This study |
| TMB3918 | W168 *amyE*::pBS1C-P$_{liaI}$ mGFPmut1_LT | This study |
| TMB4003 | W168 *amyE*::pBS1C-P$_{liaI}$ SYFP2_Eco | This study |
| TMB3919 | W168 *amyE*::pBS1C-P$_{liaI}$ sfGFP_Snp (RFC25) | This study |
| TMB3920 | W168 *amyE*::pBS1C-P$_{liaI}$ mCherry_Bsu (RFC25) | This study |
| TMB3921 | W168 *amyE*::pBS1C-P$_{liaI}$ SYFP2_Eco (RFC25) | This study |

Table S2: Primers used in this study

| Number | Sequence 5'-3' [1] | Source / Name |
|---|---|---|
| TM4067 | GATC*GGTCTC* TAATTGAGAAGGCCAAAAAACTGCTGC | This study |
| TM4068 | GATCGAATTCTATTCGATAAGCTTGGGATCC | This study |
| TM4069 | GATC*GGTCTC*TAATT G AGATTGGCCAAAGCAGAAAG | This study |
| TM4070 | GATCGAATTCTATCGTTTTCCTTGTCTTCATC | This study |
| TM4071 | GATC*GGTCTC*TAATTGGCCGACTTTAGATATTTTCGTTAT | This study |
| TM4072 | GATCGAATTCTACAGATGCATTTTATTTCATATAG | This study |
| TM4105 | GATCGGGCCCTATGAGATAATGCCGACTGTAC | This study |
| TM4106 | GATCGCTAGCTGCCTCCTAAATTTTTATCTAAAGTG | This study |
| TM4107 | GATCGGGCCCCTAAAACAATTCATCCAGTAAAATATA | This study |
| TM4108 | GATCGCTAGCATGGCTAAAATGAGAATATCACC | This study |
| TM4185 | GATCGGGCCCCAGCTCCAGATCCTCTACGC | This study |
| TM4186 | GATCGCTAGCGTTGAACTAATGGGTGCTTTAGTTG | This study |
| TM4187 | GATCGGGCCCCGATACAAATTCCTCGTAGGCG | This study |
| TM4188 | GATCGCTAGCCCCAGCGAACCATTTGAGGTG | This study |
| TM4250 | GCTAGCCTATGGTCGTTTTACAACGTGAC | This study |
| TM4251 | GAAGATCGCACTCCAGCCAG | This study |
| TM4258 | GATCTTCCCCATCGGTGATG | This study |
| TM4259 | CTCATGTTTGACAGCTTATCATC | This study |
| TM4302 | GATCGCTAGCGATCCTTTAACTCTGGCAACCC | This study |
| TM4303 | GATCGGGCCCGCCGACTGCGCAAAAGACATAATC | This study |
| TM4304 | GATCGCTAGCGAACTAATGGGTGCTTTAGTTG | This study |
| TM4305 | GATCGGGCCCCAACGGCCTCAACCTACTAC | This study |
| TM4309 | CCCTTATTATCAAGATAAGAAAG | This study |
| TM4310 | CAGCCTAAACGGATATCATC | This study |
| TM4317 | GATCCTGCAGAGGAGGCATATCAAATGAAC | This study |
| TM4318 | GATCGTCGACTTATAAAAGCCAGTCATTAGGC | This study |
| TM4325 | AATTCGCGGCCGCTTCTAGA**AGGAGG** | This study |
| TM4326 | CTAG**CCTCCT**TCTAGAAGCGGCCGCG | This study |
| TM4327 | AATTCGCGGCCGCTTCTAGA**GAGGAG** | This study |
| TM4328 | CTAG**CTCCTC**TCTAGAAGCGGCCGCG | This study |
| TM4329 | AATTCGCGGCCGCTTCTAGA**AGGAGA** | This study |
| TM4330 | CTAG**TCTCCT**TCTAGAAGCGGCCGCG | This study |
| TM4331 | AATTCGCGGCCGCTTCTAGA**GGAGAG** | This study |
| TM4332 | CTAG**CTCTCC**TCTAGAAGCGGCCGCG | This study |
| TM4333 | AATTCGCGGCCGCTTCTAGA**AGAGGA** | This study |
| TM4334 | CTAG**TCCTCT**TCTAGAAGCGGCCGCG | This study |
| TM4861 | CCGTAGGCGCTAGGGACCTC | This study |
| TM4821 | AATTCGCGGCCGCAT | This study |
| TM4822 | CTAGATGCGGCCGCG | This study |
| TM4823 | AATTCAAAAAAAAAT | This study |
| TM4824 | CTAGATTTTTTTTTG | This study |
| TM4957 | GATCGAATTCGCGGCCGCTTCTAGATAAGGAGGTCAAAAATGTCAAAAGGAGAAGAACTTTT | This study |
| TM4958 | GATCTCTGCAGCGGCCGCTACTAGTATTTATAAAGTTCGTCCATACCG | This study |

| | | |
|---|---|---|
| TM4959 | GATCGAATTCGCGGCCGCTTCTAGATAAGGAGGTCAAAAATGAGCGAACTGATCAAAGAG | This study |
| TM4960 | GATCTCTGCAGCGGCCGCTACTAGTAATTCAGTTTATGACCCAGCTTG | This study |
| TM4961 | GATCGAATTCGCGGCCGCTTCTAGATAAGGAGGTCAAAAATGAGCGAACTGATCAAAGAAA | This study |
| TM4962 | GATCTCTGCAGCGGCCGCTACTAGTAATTCAGTTTATGGCCCAGTTTT | This study |
| TM4963 | GATCGAATTCGCGGCCGCTTCTAGATAAGGAGGTCAAAAATGGTTTCAAAAGGCGAAGAA | This study |
| TM4964 | GATCTCTGCAGCGGCCGCTACTAGTACTTATAAAGTTCGTCCATGCC | This study |
| TM4965 | GATCGAATTCGCGGCCGCTTCTAGATAAGGAGGTCAAAAATGGTGAGCAAGGGCGAGG | This study |
| TM4966 | GATCTCTGCAGCGGCCGCTACTAGTACTTGTACAGCTCGTCCATGC | This study |
| TM4967 | GATCGAATTCGCGGCCGCTTCTAGATAAGGAGGTCAAAAATGGTTAGCAAAGGCGAAG | This study |
| TM4968 | GATCTCTGCAGCGGCCGCTACTAGTATTTATACAGTTCATCCATGCCC | This study |
| TM4969 | GATCGAATTCGCGGCCGCTTCTAGATAAGGAGGTCAAAAATGGTTTCCAAGGGCGAGG | This study |
| TM4970 | GATCTCTGCAGCGGCCGCTACTAGTATTTGTACAGCTCATCCATGCC | This study |
| TM4971 | GATCGAATTCGCGGCCGCTTCTAGATAAGGAGGTCAAAAATGGTTAGCAAAGGCGAAG | This study |
| TM4972 | GATCTCTGCAGCGGCCGCTACTAGTATTTATACAGTTCATCCATTCCG | This study |
| TM4973 | GATCGAATTCGCGGCCGCTTCTAGATAAGGAGGTCAAAAATGAGTAAAGGAGAAGAACTTTT | This study |
| TM4974 | GATCTCTGCAGCGGCCGCTACTAGTATTTGTAGAGCTCATCCATGC | This study |
| TM4975 | GATCGAATTCGCGGCCGCTTCTAGATAAGGAGGTCAAAAATGAGCAAAGGCGAAGAAC | This study |
| TM4976 | GATCTCTGCAGCGGCCGCTACTAGTATTTATACAGTTCATCCATGCCA | This study |
| TM4977 | GATCGAATTCGCGGCCGCTTCTAGA TAAGGAGGTCAAAAATGGCCGGCTCAAAAGGAGAAGAACTTTTTAC | This study |
| TM4978 | GATCTCTGCAGCGGCCGCTACTAGTATTAACCGGTTTTATAAAGTTCGTCCATACCG | This study |
| TM4979 | GATCGAATTCGCGGCCGCTTCTAGA TAAGGAGGTCAAAAATGGCCGGCGTTAGCAAAGGCGAAGAGG | This study |
| TM4980 | GATCTCTGCAGCGGCCGCTACTAGTATTAACCGGTTTTATACAGTTCATCCATTCCG | This study |
| TM5062 | GATCATGCATGATCCTTGTACAGCTCGTCCATGCCGAGAGTG | This study |
| TM5355 | GAATTCGCGGCCGCTTCTAGATAAGGAGGTCAAAAATGGCCGGCGTTAGCAAG | This study |
| TM5356 | GATCTCTGCAGCGGCCGCTACTAGTATTAACCGGTTTTATACAGCTCATCCATACCC | This study |
| TM5364 | GAAT TCGCGGCCGCTTCTAGATAAGGAGGTCAAAAATGGTTAGCAAGGGCGAAGAAC | This study |
| TM5365 | GATC TCTGCAGCGGCCGCTACTAGTATTATTTATACAGCTCATCCATACCC | This study |
| TM2889 | TGCCACCTGACGTCTAAG | pSB1C3 seq fwd |
| TM2890 | ATTACCGCCTTTGAGTGA | pSB1C3 seq rev |
| TM3081 | GGCAACCGAGCGTTCTG | pBS2E seq fwd |
| TM3082 | CTGACAGCGTTTCGATCC | pBS2E seq rev |

| | | |
|---|---|---|
| TM2262 | GAGCGTAGCGAAAAATCC | pBS3C*lux*checkrev |
| TM2263 | GAAATGATGCTCCAGTAACC | pBS3C*lux*checkrev |
| TM3054 | ATGAATCGGCCAACGCG | RFP-cassette front rev |
| TM3055 | TTCGGGTGGGCCTTTCTG | RFP-cassette back fwd |
| TM0498 | GCCGGTATAAAGGGACCACC | kan-Sonde fwd |
| TM0138 | CGATACAAATTCCTCGTAGGCGCTCGG | kan-rev |
| TM0139 | CAGCGAACCATTTGAGGTGATA GGGATCCTTTAACTCTGGCAACCCTC | mls-fwd |
| TM2505 | CTGATTGGCATGGCGATTGC | pBS3C*lux* sacA front check fwd |
| TM2506 | ACAGCTCCAGATCCTCTACG | pBS3C*lux* sacA front check rev |
| TM2507 | GTCGCTACCATTACCAGTTG | pBS3C*lux* sacA back check fwd |
| TM2508 | TCCAAACATTCCGGTGTTATC | pBS3C*lux* sacA back check rev |
| TM0718 | AATAGCGACGGAGAGTTAGG | *cat*-check-fwd |
| TM0719 | GGCCTATCTGACAATTCCTG | *cat*-check-rev |
| TM3137 | CCCAGTCACGTTGTAAAACG | pAC6 BS rev |
| TM0749 | AAAGGTCATTGTTGACGCGG | *amyE*-check rev |
| TM0010 | CTTCGCTATTACGCCAGCTGG | *lacZ*-check rev |
| TM3014 | CGCCGGTGATGCCTGCCACGATGCGTCCGGC | pDG1662-NgoMIV-3-mut rev |
| TM0137 | CAGCGAACCATTTGAGGTGATAGG | *kan*-fwd |
| TM0138 | CGATACAAATTCCTCGTAGGCGCTCGG | *kan*-rev |
| TM1035 | GGAAGGGCGATCGGTGCGGG | pPP2 check-rev |
| TM1038 | CGAGCGCCTACGAGGAATTTGTAT CGACCACTATGATTGGTGATGTGGAGG | hliaF do-fwd/rr03 up-fwd |
| TM3027 | GTAAAACGACGGCCAG | M13 fwd (-20) |
| TM2139 | GTTTTCCCAGTCACGAC | pRV300.Fw |
| TM3681 | GCGGCAACCGAGCGTTC | pSEVA PS2 |
| TM1225 | ATCGGTGCGGGCCTCTTCGC | pKT25 rev |
| TM4476 | CTCCTCTTTCTCTAGTATGTGTG | XylRPxyl check rev |
| TM4477 | CAATTCCCAGCATCCTCG | XylRPxyl check fwd |
| TM2557 | CTGAAATTGATCCTCCAAAC | pAX01-sacI.selfligated-fwd |
| TM3163 | GGAGCCGGTGAGCGTGGGTCACGCGGTATCATTGCAGC | pAH_BsaI_7622_mut_fwd |
| TM3164 | GCTGCAATGATACCGCGTGACCCACGCTCACCGGCTCC | pAH_BsaI_7622_mut_rev |
| TM3159 | AGGAGG GCTAGCCTATGAAATTTGGAAACTTTTTGC | pAH_SD_NheI_fwd |
| TM3160 | GGTCTCCAATTTGTCGACCTTACTAGGACACC | paH_BsaI_EcoRI_rev_P |
| TM3854 | GATCAATTGGAGACCGCGCAACGCAATTAATGTGAG | lacZalpha_BsaI_E_fwd |
| TM3855 | GATCGCTAGCTATAAACGCAGAAAGGCCCAC | lacZalpha_NheI_rev |
| TM3168 | GACAATGCTAGTAATTACTACTTGAAATATTCGTAGAGTAACGG | pLIKE-rep-SpeImut-fwd |
| TM3169 | CCGTTACTCTACGAATATTTCAAGTAGTAATTACTAGCATTGTC | pLIKE-rep-SpeImut-rev |

[1]Endonuclease recognition sites are underlined, for BsaI: recognition site shown in italic; RBS sequence indicated in bold.

Table S3: Sequences used for the Salis RBS calculator

| Name | Sequence | Start Position | Translation Initiation Rate (au) |
|---|---|---|---|
| RBS1-*lux* | AGGAGGCTAGCCTATGAAATTTGGAAACTTT | 13 | 99,564.85 |
| RBS2-*lux* | GAGGAGCTAGCCTATGAAATTTGGAAACTTT | 13 | 69,087.42 |
| RBS3-*lux* | AGGAGACTAGCCTATGAAATTTGGAAACTTT | 13 | 30,898.02 |
| RBS4-*lux* | GGAGAGCTAGCCTATGAAATTTGGAAACTTT | 13 | 29,751.76 |
| RBS5-*lux* | AGAGGACTAGCCTATGAAATTTGGAAACTTT | 13 | 30,658.04 |
| *lacZα-lux* | TTATAGCTAGCCTATGAAATTTGGAAACTTT | 13 | 1,004.96 |

| | | | |
|---|---|---|---|
| RBS1-*lacZ* | AGGAGGCTAGCCTATGGTCGTTTTACAACGT | 13 | 72,659.03 |
| RBS2-*lacZ* | GAGGAGCTAGCCTATGGTCGTTTTACAACGT | 13 | 79,074.16 |
| RBS3-*lacZ* | AGGAGACTAGCCTATGGTCGTTTTACAACGT | 13 | 12,561.07 |
| RBS4-*lacZ* | GGAGAGCTAGCCTATGGTCGTTTTACAACGT | 13 | 17,336.85 |
| RBS5-*lacZ* | AGAGGACTAGCCTATGGTCGTTTTACAACGT | 13 | 7,946.72 |
| *lacZα-lacZ* | TTATAGCTAGCCTATGGTCGTTTTACAACGT | 13 | 1,804.00 |

Table S4: Wavelengths used for plate reader measurements in nm

| Protein | Excitation spectrum | | Emission spectrum | | Endpoint[1] | |
|---|---|---|---|---|---|---|
| | Excitation | Emission | Excitation | Emission | Excitation | Emission |
| mTagBFP | 300-425 | 456 | 399 | 430-600 | 399 | 456 |
| eCFP | 350-475 | 505 | 434 | 464-600 | 449 | 479 |
| sfGFP | 400-520 | 550 | 460 | 490-600 | 481 | 511 |
| mGFPmut1 | 400-520 | 550 | 460 | 490-600 | 483 | 513 |
| mEYFP | 440-540 | 570 | 460 | 490-650 | 500 | 530 |
| SYFP2 | 440-540 | 570 | 460 | 490-650 | 500 | 530 |
| mCherry | 450-600 | 630 | 550 | 580-700 | 585 | 615 |

[1]Wavelengths used for endpoint measurements may differ from the actual observed maxima of excitation and emission for a FP. This is due to minimal required distance between excitation and emission when using the plate reader.

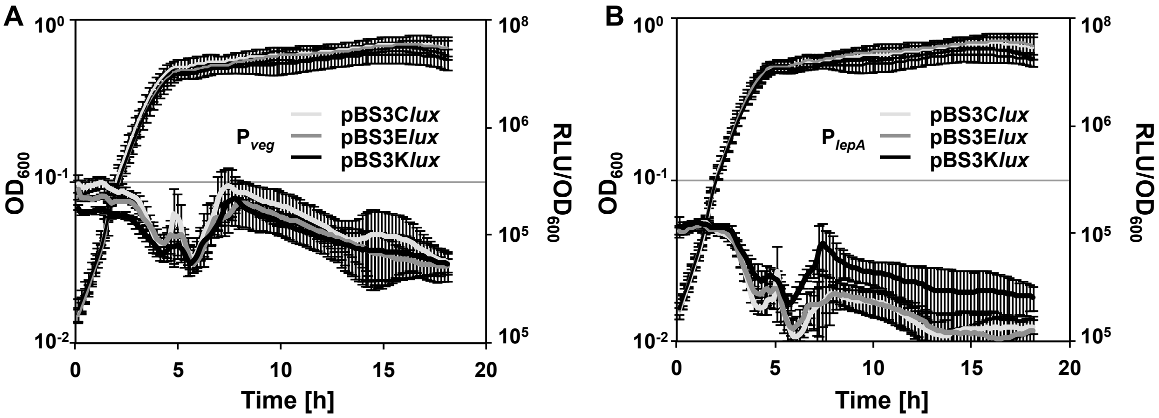


**Figure S1: Evaluation of the new *lux*-reporter vectors. (A, B)** Growth curves (left axis) and relative luminescence units divided by $OD_{600}$ (right axis, bold lines) of strains carrying either $P_{veg}$ or $P_{lepA}$ in the *lux*-reporter vectors that differ in their antibiotic resistance cassettes (TMB3090-TMB3092, $P_{veg}$, and TMB2940, TMB3088 and TMB3089, $P_{lepA}$, respectively). Measurements were performed over a time period of 18 hours and graphs show mean values and standard deviations of at least three biological replicates.

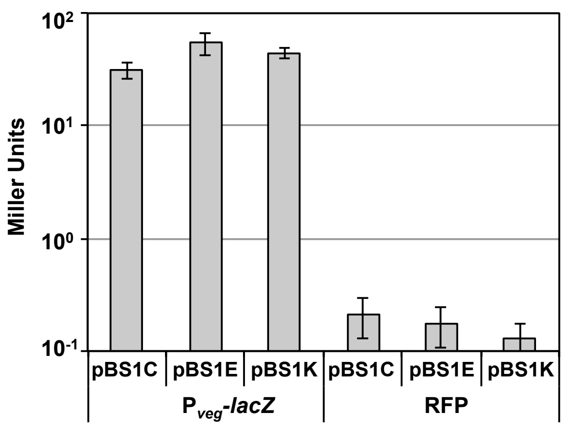


**Figure S2: Evaluation of the new empty *amyE* integrative vectors.**
Observed Miller units of the β-galactosidase assay with strains carrying $P_{veg}$ fused to *lacZ* or the empty (RFP-containing) single copy vectors, which integrate into the *amyE* locus of *B. subtilis* and differ in their antibiotic resistance cassettes (TMB3125, TMB3126 and TMB3547 for $P_{veg}$-*lacZ* constructs). Graph shows mean values and standard deviations of at least three biological replicates.

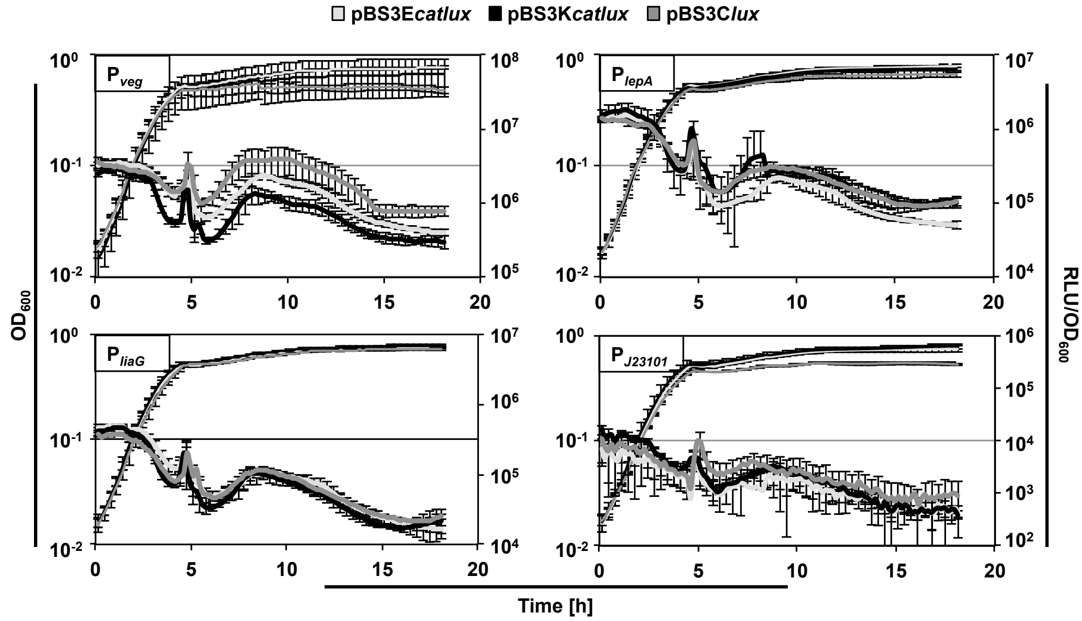


**Figure S3: Evaluation of the *catlux* promoter screening vectors. (A,B,C,D)** Growth curves (left axis) and relative luminescence units divided by $OD_{600}$ (left axis, bold lines) of strains carrying either $P_{veg}$, $P_{lepA}$, $P_{liaG}$ or $P_{J23101}$ in the *catux* promoter screening vectors which differ in their antibiotic resistance cassettes (TMB3192-TMB3199, $P_{veg}$, $P_{lepA}$, $P_{liaG}$ or $P_{J23101}$ respectively) and the original pBS3C*lux* backbone (TMB3090, TMB2940, TMB3212 and TMB3213). Measurements were performed over a time period of 18 hours and graphs show mean values and standard deviations of at least three biological replicates.

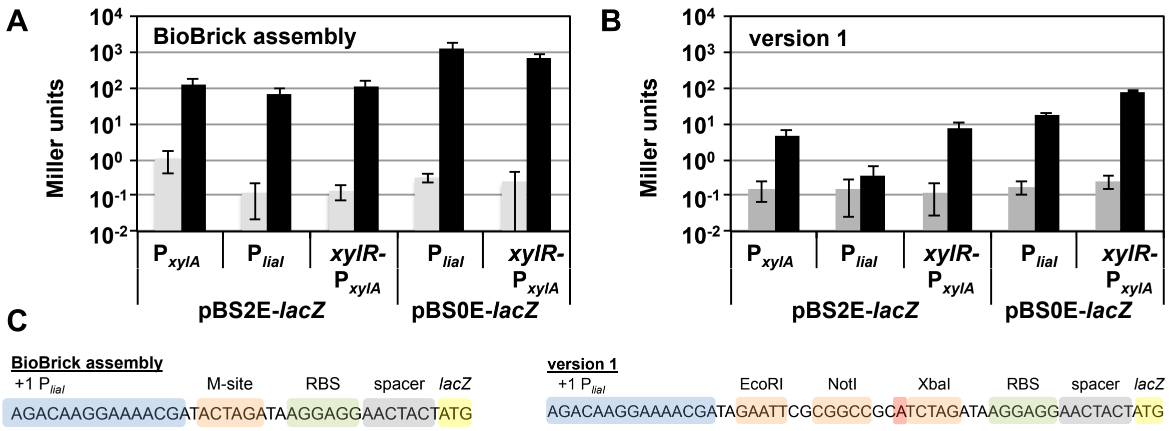


**Figure S4: Assembly layout stages of the *Bacillus* BioBrick Box 2.0 expression vectors. (A, B)** Observed Miller units of the β-galactosidase assay with strains cloned via the standard BioBrick assembly for transcriptional fusions and the expression vectors version 1 with *lacZ* used as reporter gene. Gray and black bars represent non-induced and fully induced samples, respectively. Strains TMB3497-3502 (Biobrick assembly) and TMB3532-TMB3534 together with TMB3539, TMB3540 (expression vector version 1) were grown in MCSE medium at 37°C until mid-exponential growth phase, induced with either 30 µg ml$^{-1}$ bacitracin or 0.5% xylose, and harvested 30 min or 60 min after induction, respectively. **(C)** DNA sequence of the BioBrick assembly after transcriptional fusion of two parts and the sequence layout of the expression version 1 (the in red highlighted nucleotide was changed). The lial promoter is depicted as example of the upstream promoter. (A, B) show mean values and standard deviations of at least three biological replicates.

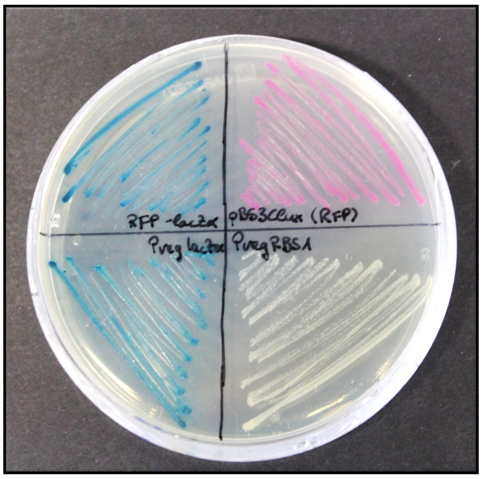


**Figure S5: Cloning stages of the RBS exchangeable screening vectors.** LB-agar plate spiked with X-gal, displaying the four stages of the RBS cloning procedure. Up left: E. coli cells harboring the RBS exchangeable empty vector. Up right: shows E. coli carrying the original pBS3Clux empty vector, with RFP in the MCS. Bottom left: shows cells with vector carrying a promoter and lacZα still present, indicating no RBS. Bottom left: E. coli harboring the vector, promoter and an RBS, resulting in white colonies.

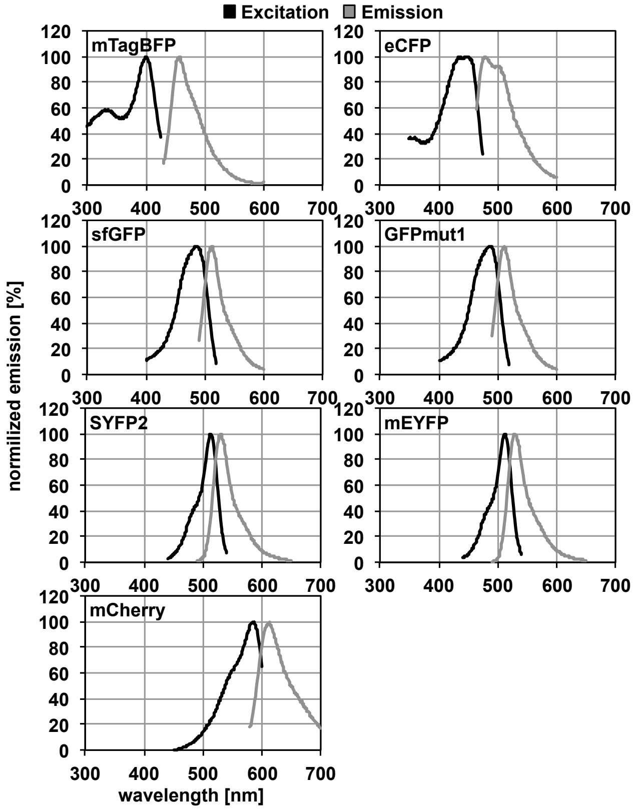


**Figure S6: Excitation and emission spectra of the *Bacillus* BioBrick Box 2.0 fluorescent proteins.** Recorded excitation (black lines) and emission (gray lines) wavelengths are depicted for each FP. Graphs represent mean values of at least three biological replicates. The corresponding strains (TMB3909, TMB3911, TMB3912, TMB3914, TMB3916-TMB3917 and TMB4003) carry the genes encoding the respective FP under control of the bacitracin-inducible promoter $P_{liaI}$. Cells were grown to exponential phase and expression of the FPs was induced by addition of bacitracin (final concentration 30 µg $ml^{-1}$) for 75 min to allow proper folding of the FPs. After induction, cells were harvested and washed with PBS and all measurements were performed in a final volume of 200 µl in 96 well plates using a Synergy™ NeoalphaB plate reader. Values were corrected against auto fluorescence of the wild type and normalized to the maxima of each measurement.

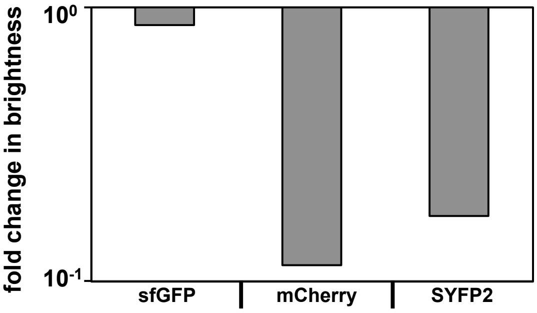


**Figure S7: Fold change in measured fluorescence of different RFC standards.** Fold change of fluorescence between strains harboring the same FP only differing in the RFC standard they are in (TMB3909, TMB3916 and TMB4003 fulfilling the RFC10 standard and TMB3919-TMB3921 complying the RFC25 standard). Cells carry the genes encoding the respective FP under control of the bacitracin-inducible promoter $P_{liaI}$. Cells were grown to exponential phase and expression of the FPs was induced by addition of bacitracin (final concentration 30 µg $ml^{-1}$) for 75 min to allow proper folding of the FPs. After induction, cells were harvested and washed with PBS and all measurements were performed in a final volume of 200 µl in 96 well plates using a Synergy™ NeoalphaB plate reader. Values were corrected against auto fluorescence of the wild type and normalized to the maxima of each measurement.

## Supplement text 1: Comment on the design aspects regarding the *Bacillus* BioBrick Box 2.0 expression vectors.

We performed translation initiation rate (TIR) predictions of both versions, 3A and expression vectors (EVs), with the downstream reporter gene *lacZ*. We found that the presence of the BioBrick prefix (in the EVs versions) interferes with the ribosome binding site of the *lacZ* gene due to the formation of secondary structures and thus potentially prevents functional expression, which could explain the too low measured outputs of the EVs. In order to test this hypothesis, we designed two new versions of our expression vectors by changing nucleotides within the BioBrick prefix. In the case of EV version 1, we changed a thymine between the NotI and the XbaI sites to an adenine, aiming at preserving all restriction enzyme sites of the RFC10 standard but decreasing the secondary structure formation. In the second case (version 2) we removed the entire GC-rich NotI site and replaced it by a poly-adenine stretch of the same length (figure 2). Re-evaluation of both expression vector versions and comparison to the constructs cloned via 3A assembly confirmed our assumption. While expression vectors of version 1 were working notably better compared to the original EVs (~6 miller units for single and ~60 for multi copy versions), the 3A assembly cloned constructs showed again much higher output (supplement figure S4). Only the EV version 2 could regain the same expression-levels as observed with the standard BioBrick assembly (figure 2D, S4). The single copy EV version 2 gave measured outputs of about 70 miller units and the multi copy vectors approximately 1200 miller units. Despite the fact that we could achieve a recovery in terms of output, additional aspects are of importance when it comes to working with the expression vectors. First, in the case for EV version 2 the insertion of the gene of interest into the MCS should be performed via XbaI and PstI in order to preserve the sequence layout prior to the XbaI site, (the insertion via XbaI and SpeI is possible, but not recommended because both restriction enzymes give rise to compatible overhangs which leads to self-ligation and non-directional cloning). Second, the downstream inserted CDS plays a major role on the functionality of the expression vectors, which is plausible, since our observations are sequence dependent. With experiences in our own lab routine (data not shown) together with previous investigation regarding this topic[1,2], we recommend to perform predictions of secondary structure formation and translation initiation rates of the desired. As a third aspect, we like to point out recent studies, which specifically focus in the effects of genetic context on gene expression[3,4]. We believe a general implementation of small standardized elements to fine tune gene expression and increase the reliability of composite parts should be a main future goal of the iGEM community.

1. Liebeton, K., Lengefeld, J. & Eck, J. The nucleotide composition of the spacer sequence influences the expression yield of heterologously expressed genes in *Bacillus subtilis*. *Journal of Biotechnology* **191,** 214–220 (2014).

2. Band, L. & Henner, D. J. *Bacillus subtilis* requires a ‘stringent’ Shine-Dalgarno region for gene expression. *DNA* **3,** 17–21 (1984).
3. Lou, C., Stanton, B., Chen, Y.-J., Munsky, B. & Voigt, C. A. Ribozyme-based insulator parts buffer synthetic circuits from genetic context. *Nat. Biotechnol.* **30,** 1137–1142 (2012).
4. Mutalik, V. K. *et al.* Precise and reliable gene expression via standard transcription and translation initiation elements. *Nat. Methods* **10,** 354–360 (2013).
